# The effects of negative economic shocks at birth on adolescents’ cognitive outcomes and educational attainment in Malawi

**DOI:** 10.1016/j.ssmph.2022.101085

**Published:** 2022-04-07

**Authors:** Fabrice Kämpfen, Fatima Zahra, Hans-Peter Kohler, Rachel Kidman

**Affiliations:** a School of Economics, University College Dublin, Ireland; b Population Studies Center, University of Pennsylvania, Philadelphia, PA, USA; c GIRL Center, Population Council, Washington, D.C, USA; d Department of Family, Population and Preventive Medicine, Stony Brook, NY, USA

**Keywords:** *Economic shock*, *Early life adversity*, *Cognitive outcomes*, *Schooling*, *Investments in education*, *Adolescents*, *Malawi*

## Abstract

*We provide new evidence of the association between moderate negative economic shocks* in utero *or shortly after birth and adolescents’ cognitive outcomes and educational attainment in Malawi. This is one of the first studies to analyze the effect of not one, but multiple moderate negative economic shocks in a sub-Saharan African (SSA) low-income country (LIC). This focus is important as multiple economic shocks in early life are more representative of the experiences of adolescents in LICs. Combining data on adolescents aged 10–16 from the Adverse Childhood Experiences (ACE) project with the Malawi Longitudinal Study on Families and Health (MLSFH)* (*N* = 1, 559)*, we use linear and probit regression models to show that girls whose households experienced two or more economic shocks in their year of birth have lower cognitive scores, which are measured using working memory, reading and mathematical tests. Girls also have lower educational attainment, conditional on age. These effects are gendered, as we do not observe similar effects among boys. Overall, our results point to lasting effects of early-life adversity on adolescents, and they highlight that, even in a LIC context where early-life adversity is common, policymakers need to intervene early to alleviate the potential long-term educational impacts of* in utero *or early life shocks among girls.*

## 1 Introduction

Prenatal and early childhood conditions are critical for long-term human capital development (Almond & Currie, 2011; Georgiadis et al. 2017). Prior studies have identified the human capital effects of these conditions, showing that both extreme and subtle shocks in utero and during early childhood can have lasting effects on later educational attainment, test scores, and child health (Almond et al. 2018; Brown, 2018; Cook et al. 2019; Lee, 2014). These negative shocks can affect children through both biological and social pathways that determine educational and cognitive outcomes. For instance, prenatal and postnatal malnutrition can damage brain development (Levitsky & Strupp, 1995). In addition, it may lower parental investments in maternal or child nutrition and thus affect children’s long-term cognitive outcomes (Almond et al. 2018; Wolf & McCoy, 2019). Together, these pathways help form the environment within which the cognitive development of the fetus and young children takes place, and set a foundation for later educational achievement.

Most research from low-income countries (LICs) has examined extreme climate shocks, famine, and violence, showing detrimental effects on test scores and educational attainment (Ampaabeng & Tan, 2013; Millett & Shah, 2012; Rosales, 2013). There is scarce evidence on how multiple, relatively frequently occurring negative shocks—which are more common to households in these contexts—affect these outcomes. Few studies in sub-Saharan Africa have shown how such shocks in utero and early childhood influence adolescents’ educational outcomes (Beshir & Maystadt, 2020), despite the fact that repeated exposure to moderate shocks is a much more common early-life experience in LICs than exposure to severe shocks.

Using a rare LIC dataset that links shocks and household conditions in the year of birth with Malawian adolescents’ cognitive and educational outcomes, we contribute to literature on the relationship between shocks in the year of birth and adolescent educational outcomes by focusing on four research questions that, to date, have received scant attention in LICs: 1. Do multiple negative shocks experienced in the year of birth impact adolescent educational attainment; age for grade progression; and reading, math, and working memory test scores? 2. Do we observe differences in coefficient size for adolescents’ educational outcomes when shocks are restricted to those that affect the entire community and are more plausibly exogenous than other household shocks? 3. Do the effects of these shocks on adolescent outcomes differ by gender? 4. Do nutritional investments (proxied by anthropometric measures) and education investments mediate the relationship between economic shocks and adolescents’ cognitive outcomes?

Overall, our analyses show that multiple shocks in the year of birth adversely affect girls’ educational outcomes. Specifically, girls who experience two or more economic shocks are more likely to be unable to read sentences in Chichewa, unable to recall numbers (working memory), have lower overall math scores, educational attainment, and composite summary index scores compared to girls who experienced no shock in their year of birth. We find similar results for economic shocks that affect the entire community and are more plausibly exogenous than other household shocks. Importantly, our results reveal gendered associations of early-life adversity on adolescent cognitive and educational outcomes, given that we do not find similarly strong associations among boys. This effect seems to be driven by gendered compensating behaviors by households in response to shocks. Notably, we find evidence of greater household investment in boys’ education in response to shocks.

## 2 Background: limited evidence on the long-term effects of early-life adversity on adolescents in LICs

The fetal origins hypothesis states that the prenatal environment can affect the fetus, with both short and long-term consequences for health outcomes (Barker, 1990). Prior studies have expanded on this, hypothesizing the effects of both prenatal and postnatal investments on long-term human capital development (Almond et al. 2018; Heckman, 2007). This is predicated on the assumption that the development of human capital is linked across the life-course. A dearth of investment during this critical period, for instance as a result of negative shocks that adversely affect a household, can be harmful for outcomes measured a decade or more later in life. Thus, children with unfavorable prenatal or early childhood conditions may not only suffer worse outcomes in later periods, they may also have lower returns on the investments made in them due to early disadvantages (Almond & Currie, 2011; Heckman, 2007).

Shocks in the gestation period (prenatal) and early childhood (postnatal) can affect children through both biological and social pathways (Almond et al. 2018), but disentangling these pathways is often difficult. Biologically, prenatal malnutrition can alter brain neural receptor pathways through permanent effects on the hippocampus and cerebellum (Levitsky & Strupp, 1995). In addition, negative prenatal shocks can result in adverse birth outcomes like low birth weight, which has been linked to low educational attainment and poor test scores in childhood and adulthood (Almond & Currie, 2011; Almond et al. 2018). Postnatal malnutrition can also inflict damage on brain development (Levitsky & Strupp, 1995; Uauy & Dangour, 2006). However, biological effects on cognitive development may not manifest until a later period, suggesting that the effects of prenatal and postnatal shocks may be irregular over age (Heckman, 2007).

Shocks may also affect educational outcomes through social pathways, with long-term implications for educational outcomes and test scores. For instance, parental preferences may determine investments in child health and education in response to a shock, affecting the timing of school enrollment and the likelihood of remaining enrolled and on track in school. Recent additions to these hypotheses pay particular attention to the role of parental investments. Linking postnatal investments in response to shocks in utero, Almond et al. (2018) hypothesize that postnatal investments in children depend on parents’ preferences, budget constraints, and constraints in production technology. In turn, these preferences, which can include gender preferences, may mitigate or worsen the long term cognitive impact of negative shocks experienced in the prenatal and early childhood periods (Becker & Tomes, 1976; Behrman et al. 1982).

Despite the reality that experiences of early-life adversity are common in LICs, the literature documenting the relationship between negative prenatal and postnatal shocks and long-term educational outcomes has several limitations. First, studies on high income countries (HICs) and middle income countries (MICs) (Aizer et al. 2016; Almond et al. 2015; Greve et al. 2017; von Hinke Kessler Scholder et al. 2014) often investigate shocks that are more relevant to HIC or MIC contexts rather than LIC contexts.

Second, LIC studies on early-life adversity have often investigated extreme negative shocks, which are important, but by their very definition, are relatively rare.^1^ Examples include: El-Nino floods in Ecuador (Rosales, 2013), famine in Ghana (Ampaabeng & Tan, 2013), genocide and war in Rwanda and Zimbabwe (Alderman et al. 2006; Bundervoet & Fransen, 2018), drought in India, Burkina Faso, and Zimbabwe (Akresh et al. 2012; Alderman et al. 2006; Hoddinott & Kinsey, 2001; Millett & Shah, 2012). Across all of these studies, the key finding is that children exposed to shocks in utero and early childhood have lower test scores and educational attainment. These effects can also persist across generations. For instance, Tafere (2016) finds intergenerational effects of famine and shows that the children of Ethiopian mothers who were exposed to famine between ages 0–3 are more likely to have lower test scores, educational attainment, and poorer health. A rare example of a study that has focused on moderate shocks is from Ethiopia (Beshir & Maystadt, 2020) and shows that exposure to seasonal food insecurity experienced in utero results in lower math scores at age 8 and 12.

Third, a further limitation of the existing literature is its emphasis on a single positive or negative shock in utero. Studies that examine more than one shock typically analyze whether a negative shock can be compensated by a positive shock, usually a conditional cash transfer (Adhvaryu et al. 2018; Aguilar & Vicarelli, 2011; Duque et al. 2018). To our knowledge, no previous studies examine the impact of multiple, moderate negative shocks experienced in utero and early childhood on adolescents’ educational and cognitive outcomes. This is important to investigate in sub-Saharan African low-income countries, such as Malawi, where households are likely to experience multiple shocks related not only to income, but also excess adult mortality due to epidemics like HIV. Furthermore, previous studies that have distinguished between the effects of shocks to the household and shocks to the entire community have focused on school enrollment (Hyder et al. 2015), and not the long-term educational and cognitive outcomes of children who experience these shocks in the year of birth. Community level shocks, which are more likely to be exogenous, might make it difficult for households to buffer a shock through the support of their neighbors or social network, thus causing greater severity in detrimental impacts on children’s outcomes.

Although our study focuses on negative household shocks, there are many other conditions and experiences during early childhood that may positively or adversely affect future educational and cognitive outcomes. As examples, these can include shocks to child health and nutrition (Almond et al. 2018), adverse childhood experiences (Guinosso et al. 2016), and early stimulation, nutrition, or economic interventions (Tanner et al. 2015).

Fourth, while previous studies have tested for gendered effects, there has been limited attention on the gendered mechanisms through which these effects might manifest. For instance, parents’ gender preferences may influence the investment choices they make for their sons and daughters, and these preferences may be reinforced when making investment decisions after experiencing negative shocks. Related evidence from sub-Saharan Africa is particularly scarce. Most evidence of general gender bias in parental educational investment comes from South Asia (Azam & Kingdon, 2013; Kaul, 2018). In sub-Saharan Africa, there is mixed evidence of gender bias in intra-household allocation of resources towards health and education (Haddad & Reardon, 1993; Hadley et al. 2008; Sauerborn et al. 1996, pp. 131–145). A recent study from Ethiopia finds that boys exposed to seasonal food insecurity in utero are more likely to have low math scores at age 12, compared to girls. However, these differences cannot be explained by parental education and health investments (Beshir & Maystadt, 2020). In addition, these studies do not consider how parents’ informal social networks, which may be an important resource when households face budget constraints, reinforce or mitigate gender bias when providing support.

## 3 Data and measures

Our analyses are based on the Adverse Childhood Experiences (ACE) project (Kidman et al. 2020) of the Malawi Longitudinal Study of Families and Health (MLSFH) (Kohler et al. 2015). This MLSFH ACE project focuses on ACEs and transitions to adulthood, collecting data in rural areas in three districts in Malawi (Mchinji, Rumphi and Balaka).^2^ In 2017/18 (and a new round in 2021 that was not yet available for the analyses in this paper), data on MLSFH ACE adolescents were linked to prior MLSFH data for the adolescents’ parents, dating back to 1998. The initial MLSFH cohort was established using a cluster random sampling strategy (Mchinji and Rumphi) and by drawing a subset of an earlier representative population survey (Balaka) to represent the rural population of Malawi, where the majority (85%) of Malawians live in conditions that are similar to those in the rural areas of other countries with high HIV prevalence. The initial sample characteristics closely matched the characteristics of the rural population of the 1996 Malawi Demographic and Health Survey (DHS) (see Kohler et al. (2015).^3^ This study draws on the currently available first wave of MLSFH ACE adolescent surveys collected in 2017–18, when respondents were 10–16 years old.^4^ Importantly, the MLSFH ACE data provide comprehensive measures on a range of cognitive outcomes among adolescents, which we use as the main dependent variables in our study. The data also has measures on a number of other adolescent experiences including health, violence, and relationships with caregivers.

At least one parent (or household member)^5^ of the 2017–18 ACE adolescent respondent was previously surveyed in 2008 or 2010, when they were asked to report economic shocks that they or their household experienced over previous years.^6^ We match adolescent’s year of birth as reported in the ACE study to their household’s information collected in 2008 and 2010, which includes economic shocks reported between 2003 and 2008.^7^

We only have information about economic shocks for the period between 2003 and 2010. Thus, out of the 2,089 adolescents that were interviewed as part of the ACE study, 273 adolescents were excluded from the analysis because they were born in 2001 or 2002. Moreover, we excluded a further 257 adolescents who were born between 2003 and 2007, and whose households were surveyed only in 2010 and thus had no shock data for the year of their birth. Our final sample includes 1,559 adolescents, for whom we have information about whether their household experienced economic shocks in the year that they were born.

**Cognitive measures:** The MLSFH ACE data provide several measures of cognitive outcomes that encompass three different domains: literacy, mathematical skills, and working memory. We derive two outcome variables for each of these domains: one that characterizes the total score (continuous variable) obtained in the various tests, and another that takes the value 1 if adolescents obtain a score of 0 in a specific domain, and 0 otherwise. These dichotomous variables allow to explore the effects of economic shocks at birth at the lower end of the various cognitive scores. We provide details for tests in each domain in Appendix A. Reading and math tests are broadly representative of cognitive outcomes used in other studies (Beshir & Maystadt, 2020; Millett & Shah, 2012; Tafere, 2016), whereas working memory is not a common measure of cognitive development used in this literature. In addition, measures of IQ such as the Raven test (Ampaabeng & Tan, 2013) while common, are not available for examination in our study.

For other measures of education, we also included schooling attainment and on-time progression in school. For schooling attainment, we measure highest grade attained. For on-time progression in school, we used a dichotomous variable that takes the value 1 if adolescents are at least 3 years behind the expected grade for their age, and 0 otherwise.^8^

Our set of outcome variables is therefore constituted of four continuous and four dichotomous variables. To deal with issues of overrejection of the null hypothesis due to multiple inference, we follow Anderson (2008) and create summary indices for each of these two sets of outcomes. This approach also has the advantages of providing an estimate of the overall effect of the economic shocks and of potentially being “*more powerful than individual-level tests—multiple outcomes that approach marginal significance may aggregate into a single index that attains statistical significance*” (Anderson, 2008, p. 1484).^9^ In the results section below, we first present the associations between economic shocks experienced at birth and the two summary indices, and then detail these associations for each of our four types of outcome variables.

**Economic shocks:** In the 2008 and 2010 MLSFH surveys, respondents were asked to report economic shocks experienced by their households that ***negatively*** affected their income and/or assets. These shocks are reported in Table 1. In both survey years, respondents were asked to report the shocks they experienced, along with the year when the three most “significant” shocks occurred. In addition to the years of occurrence, they were asked whether the shock they reported affected their “own household only”, “other households as well”, “most households in the community” or “all households in the community”. We match the years of occurrence of these economic shocks to the years of birth of the ACE adolescents in our sample.

**Table 1:** Descriptive statistics of the economic shocks at birth reported.

	Count	Prevalence
− **Death or serious illness** of an adult member or someone who provides support for yourself or your family	93	0.241
− **Poor crop yields**, loss of crops due to disease or pests, or loss of livestock due to theft or disease, or loss of coupon	159	0.412
− **Loss of source of income** -such as loss of employment, business failure, someone who had been assisting the household stopped their support	63	0.163
− **Big change in price of grain** (either increase or decrease) ^*a*^	116	0.301
− **Fertilizer subsidy**	2	0.005
− **Breakup of household**, such as a divorce	24	0.062
− **Damage** to house due to fire, flood, or other unexpected event	19	0.049
− **Other**	2	0.005

*Note*: These shocks are reported by adolescents’ households as part of the MLSFH collected in 2008 and 2010. “Count” corresponds to the number of adolescents in our sample who experienced a particular economic shock at birth. “Prevalence” corresponds to the % of adolescents in our sample who experienced a particular economic shock, conditioning on experiencing a shock at birth. ^*a*^ “Big change in price of grain” can potentially represent positive or negative shocks depending on whether the household is a net consumer or producer of crops. However, the survey asks respondents whether the economic shocks they report resulted in “income loss”, “asset loss”, “loss of both” or “neither”. Our analysis is restricted to shocks that resulted in income loss, asset loss or both, i.e., negative economic shocks.

**Descriptive statistics of study population:**Table 2 presents the descriptive statistics of the 1,559 adolescents that constitute our study sample. Panel A shows basic descriptive statistics of the outcome variables we consider in our analysis. The average reading score in our sample was about 4.4, on a scale from 0 to 8. About 32% of the adolescents couldn’t read, even partially, the two sentences in Chichewa that were presented to them. On a scale ranging from 0 to 7, the average working memory score of the adolescents in our sample was about 2.5 and about 7.5% of them had a score of 0. The average math score was about 6.9, out of 12 points, and a bit more than 6% of the sample had a score of 0.

**Table 2:** Descriptive statistics of the study sample (*N* = 1, 559).

	Mean	Std. dev.	25^th^	75^th^	Obs.
***A. Outcome variables***					
Summary index - continuous outcomes	−0.071	1.001	−0.881	0.649	1557
Summary index - discrete outcomes	0.023	1.047	−0.886	0.453	1557
Reading score (sentences)	4.424	3.371	0	8	1544
Can’t read Chichewa sentences	0.315	0.465	0	1	1546
Working memory score	2.477	1.623	1	3	1278
Working memory score of 0	0.075	0.264	0	0	1278
Math score	6.853	3.505	5	10	1513
No correct math answers	0.062	0.241	0	0	1513
School attainment (years)	4.658	1.890	3	6	1557
Age for grade ⩾ 3	0.617	0.486	0	1	1450
***B. Economic shock***					
Shock at birth	0.248	0.432	0	0	1559
0 shock at birth	0.752	0.432	1	1	1559
1 shock at birth	0.194	0.395	0	0	1559
2 shocks or more at birth	0.054	0.226	0	0	1559
***C. Control variables***					
Girl	0.491	0.500	0	1	1559
Age	12.831	1.464	12	14	1559
Central region	0.305	0.460	0	1	1559
South region	0.371	0.483	0	1	1559
North region	0.325	0.468	0	1	1559
Age of the caregiver at birth	31.984	13.334	22	38	1558
Caregiver married at birth	0.872	0.335	1	1	1559
No formal education - caregiver	0.262	0.440	0	1	1559
Primary level education - caregiver	0.654	0.476	0	1	1559
Secondary level education or higher - caregiver	0.084	0.278	0	0	1559
Wealth score	−0.081	1.868	−1.318	0.800	1557

*Note*: The sample is derived from the ACE sample collected in 2017 and 2018. Economic shocks are reported by adolescent’s household as part of the MLSFH collected in 2008 and 2010. “Std. dev.” stands for standard deviation. 25^th^ and 75^th^ represent the 25^th^ and 75^th^ percentiles of the distributions, respectively.

Panel B of Table 2 shows the distribution of the economic shocks experienced by the adolescents the year of the birth. About three quarters of the adolescents in our sample experienced no economic shocks at birth, whereas about 19% and 5.4% of them experienced one and two shocks or more the year of their birth, respectively. Importantly, Appendix Table B1 shows that boys and girls do not differ in terms of the number of shocks reported during the year of birth. Moreover, when regressing the number of shocks experienced during the year of birth on a set of household and caregiver characteristics, none of the factors appear statistically significant at conventional levels (see Appendix Table B2). This holds true even when we interact all regressors with the sex of the adolescent. The tests of joint-significance reported at the bottom of the table confirm these results.

Table 1 reports the types of shocks that the adolescents experienced during their year of birth and the number of adolescents (“Count”) in our sample who were affected by these shocks at birth. The most prevalent negative shocks, which represent about 41% of the economic shocks encountered, correspond to shocks that have resulted in poor crop yields “due to disease or pests, or loss of livestock due to theft or disease, or loss of coupon”.^10^ The second most prevalent shock is “big change in price of grain (either increase or decrease)”, which represents about 30% of the shocks reported. These two shocks, which account for about 71% of the shocks, are plausibly more exogenous than others as they are more likely to be independent to the respondent’s or household’s characteristics. In fact, we show in Appendix Tables B3 and B4 that none of the adolescent or caregiver characteristics we consider in our analysis predict these two shocks at conventional significance levels. This is confirmed by the tests of joint-significance we report at the bottom of these two tables. They can thus be used to reinforce the close to causal interpretation of our effects, as we will discussed below. In order, “death or serious illness”, “loss of income”, “breakup of household”, “damage to house” and loss of “fertilizer subsidy” represent 24.1%, 16.3%, 6.2%, 4.9% and 0.5% of the experienced economic shocks, respectively. Moreover, there exists no statistical difference (at 95% confidence) in the types of shocks experienced by boys and girls in our sample (Appendix Table B1).

Finally, Panel C of Table 2 shows basic descriptive statistics of the adolescents in our sample. Slightly less than half (49.1%) of the adolescents were girls and the average age was about 12.8 years old. Adolescents were evenly distributed across our three study regions. Appendix Table B5 shows that there exists no statistical difference in the characteristics of the boys and girls in our sample, except in terms of age, where boys appear to be on average marginally younger than girls (12.,7 vs 12.9 years old, respectively). The normalized difference in age between these two groups however is equal to 0.088, which is well below the 0.25 threshold that is often taken as indicative of imbalance (Imbens & Rubin, 2015).

## 4 Analytic approach

We match ACE adolescents surveyed in 2017 and 2018 to shocks reported by their parents (or caregivers) in 2008 and 2010, to create a sample of ACE adolescents who experienced economic shocks during the year of birth. We then regress our cognitive and education measures on our main independent variable: economic shocks. We have two dichotomous measures of economic shocks. The first is coded 1 if the adolescent experienced one economic shock at birth, and 0 otherwise. The second is coded 1 if they experienced two or more economic shocks at birth, and zero otherwise.^11^ We conduct linear regressions for all continuous dependent variables, including the summary indices, reading, math, and working memory scores, and schooling attainment (measured as highest grade attained). We use probit regressions for all dichotomous dependent variables.

More specifically, the econometric model we estimate is the following:

(1) $$y_{i}=\alpha +\beta_{0}\;{One\;Shock}_{i}+\beta_{1}\;{Two\;Shocks}_{i}^{+}X_{i}^{\prime }\gamma +\varepsilon_{i},$$

where *y*_*i*_ refers to a particular cognitive outcome and *One Shock*_*i*_ and ${Two\;Shocks}_{i}^{+}$ are dichotomous variables that take the value 1 if an adolescent in our sample experiences one and two or more economic shocks at birth, respectively. Our vector of control variables *X*_*i*_ includes the age of the adolescent (dummy variables for each age in years), characteristics of the caregivers including age, marital status, educational level (no school, primary level of education, secondary level of education and higher education), and a continuous wealth index based on a set of 20 dwelling characteristics and ownership of household durable assets, constructed using first principal component analysis (Chin, 2010; Filmer & Pritchett, 1998; Hyder et al. 2015; Vyas & Kumaranayake, 2006). Wealth measures based on household asset ownership are usually used to control for *stable* household wealth characteristics (Behrman & Knowles, 1999; Thomas & Strauss, 1992). We use the most up-to-date information available at the year of birth to define these variables. In other words, information collected in wave 5 (2008), wave 4 (2006) and wave 3 (2004) was used to define these variables for children born in 2007–2008, 2005–2006 and 2003–2004 respectively. For missing cases, we use the most recent information available.^12^ In addition to these variables, all regressions include region dichotomous variables to control for any systematic differences in the three regions where fieldwork took place (Rumphi in the North, Balaka in the South and Mchinji in the central region of Malawi). Finally, because some adolescents were interviewed in 2017 and others in 2018, all our specifications include a binary variable coded 1 if the survey was conducted in 2017 vs. 2018. This year dummy captures any systematic differences and changes that might have occurred in 2018. For all our analyses, standard errors are clustered at the household level to take into account the fact that some (few) of the adolescents in our sample live in the same household.

## 5 Results

Our results show that experiencing two or more (“two^+^”) negative economic shocks at birth is negatively associated with cognitive and educational outcomes for girls, while there is no evidence for corresponding detrimental associations for boys. Specifically, Table 3 presents the associations between economic shocks at birth and our set of outcome variables. The first three columns of the top panel show that, on average, adolescents who experience two^+^ economic shocks at birth have a lower weighted average of the cognitive outcomes considered (about 0.164 points) (Column 1). This negative association is observed among girls (Column 3, *β* = -0.337, p-value = 0.031) but not among boys (Column 2). The difference in the coefficients between boys and girls is statistically significant at 90% confidence in a one-sided test (z-score = 1.586, p-value = 0.056). When looking at the summary index derived from the set of discrete outcomes (Columns 3–6), adolescents who experience two^+^ economic shocks at birth had a higher summary index (*β* = 0.302, p-value = 0.034), reflecting the negative associations that these shocks have on cognitive outcomes.^13^ Again, these associations are mainly observed among girls (*β* = 0.551, p-value = 0.011). The difference in these associations between boys and girls is statistically significant at 95% confidence in a one-sided test (z-score = -1.657, p-value = 0.049). In contrast to experiencing two^+^ economic shocks at birth, experiencing only one economic shock at birth does not have any impact on adolescents’ cognitive outcomes as measured by our two summary indices, irrespective of the sex of the adolescent.

**Table 3:** Associations between economic shocks at birth and cognitive and educational attainment outcomes.

	All (1)	Boys (2)	Girls (3)	All (4)	Boys (5)	Girls (6)
	***Summary index***					
	Continuous outcomes^*a*^			Discrete outcomes^*b*^		
1 shock at birth	0.025	0.061	−0.033	0.030	−0.020	0.088
	(0.056)	(0.077)	(0.085)	(0.066)	(0.087)	(0.103)
2 shocks or more at birth	−0.164	−0.014	−0.337*	0.302*	0.086	0.551*
	(0.103)	(0.132)	(0.156)	(0.142)	(0.179)	(0.216)
Observations	1554	792	762	1554	792	762
	***A. Reading skills***					
	Reading score			Can’t read Chichewa		
1 shock at birth	0.123	0.217	−0.047	−0.014	−0.037	0.035
	(0.204)	(0.288)	(0.300)	(0.092)	(0.124)	(0.149)
2 shocks or more at birth	−0.439	0.004	−0.925^+^	0.280^+^	0.164	0.435^+^
	(0.366)	(0.494)	(0.530)	(0.165)	(0.233)	(0.234)
Observations	1541	786	755	1543	787	756
	***B. Working memory***					
	Working memory score			Score of 0		
1 shock at birth	0.025	0.113	−0.063	0.010	−0.299	0.354
	(0.114)	(0.149)	(0.180)	(0.145)	(0.212)	(0.223)
2 shocks or more at birth	−0.295	−0.112	−0.488	0.477*	0.385	0.670*
	(0.204)	(0.246)	(0.309)	(0.218)	(0.306)	(0.336)
Observations	1276	644	632	1276	644	545
	***C. Mathematical skills***					
	Math score			Score of 0		
1 shock at birth	−0.042	−0.036	−0.108	0.143	0.054	0.320
	(0.215)	(0.301)	(0.319)	(0.137)	(0.171)	(0.228)
2 shocks or more at birth	−0.563	−0.433	−0.783	0.207	−0.158	0.748*
	(0.377)	(0.527)	(0.536)	(0.214)	(0.308)	(0.311)
Observations	1510	770	740	1510	770	678
	***D. Schooling***					
	Educational attainment			Age for grade ⩾ 3 yrs		
1 shock at birth	0.017	0.109	−0.120	−0.024	−0.027	−0.008
	(0.087)	(0.113)	(0.134)	(0.096)	(0.129)	(0.147)
2 shocks or more at birth	−0.114	0.200	−0.413^+^	0.100	0.007	0.260
	(0.161)	(0.205)	(0.246)	(0.177)	(0.255)	(0.258)
Observations	1554	792	762	1447	738	709

*Note*: Standard errors in parentheses clustered at the household level (^+^*p* < 0.10, *p < 0.05, **p < 0.01). The sample is derived from the ACE sample collected in 2017 and 2018. All regressions control for age (in years) and region fixed effects, age and marital status of the caregiver at birth, educational level of the caregiver (no school, primary level of education, secondary level of education and higher of education), a continuous wealth index of the household and sex of the adolescent. ^*a*^: Lower values indicate worse cognition/education outcomes. ^*b*^: Higher values indicate worse cognition/education outcomes.

Panels A, B, C and D show the corresponding estimates by breaking down the summary indices into their four components; reading skills, working memory, mathematical skills and schooling, respectively.^14^ Across the four panels, we observe that adolescents who experience two^+^ economic shocks at birth consistently have lower cognitive scores relative to those who do not experience any shock. While not always precisely estimated when pooling boys and girls together, these associations are consistently negative, irrespective of whether we consider the continuous or discrete measures of cognitive outcomes. Similar to summary indices, these associations are observed only among girls, where five out of eight coefficients are statistically significant (at least at 90% confidence).

Overall, experiencing a single economic shock during the year of birth does not seem to affect the cognitive outcomes of adolescents in our sample. However, experiencing two^+^ economic shocks at birth is associated with adolescents’ cognitive outcomes, but these associations are statistically significant only among girls.

Economic shocks can be particularly detrimental when they affect entire communities, since this limits households’ ability to buffer the impact of shocks by seeking social support from their neighbors. Among the economic shocks reported by respondents in 2008 and 2010, two are “plausibly exogenous” in that they are more likely to not be related to individual and household characteristics or behaviors. “Poor crop yields” and “big change in price of grain” are likely to be beyond an individual household’s control and hence largely exogenous (see Appendix Tables B3 and B4). We therefore restrict our economic shock variable to these two “plausibly exogenous” shocks to strengthen the causal interpretation of our estimates. As an additional check for exogeneity, respondents are asked whether the shocks they report affected other households in their community. We are therefore able to restrict these two shocks to those that affected other households in the community in order to reinforce the causal interpretation of our estimates (because these restrictions reduce the number of shocks reported by the respondents, we are not able to differentiate between adolescents who experienced one or two^+^ exogenous shocks at birth and hence present results in which we combine adolescents who experience one or more exogenous shocks in the same category). Table 4 presents the results for these plausibly exogenous shocks on our dependent variables.

**Table 4:** Associations between economic shocks at birth and cognitive and educational attainment outcomes using plausible exogenous shocks.

	All (1)	Boys (2)	Girls (3)	All (4)	Boys (5)	Girls (6)
	***Summary index***					
	Continuous outcomes^*a*^			Discrete outcomes^*b*^		
Exogenous shock at birth	−0.111^+^	0.012	−0.272*	0.193*	0.034	0.415**
	(0.066)	(0.085)	(0.105)	(0.089)	(0.112)	(0.142)
Observations	1554	792	762	1554	792	762
	***A. Reading skills***					
	Reading score			Can’t read Chichewa		
Exogenous shock at birth	−0.345	−0.032	−0.856*	0.240*	0.125	0.436**
	(0.244)	(0.328)	(0.367)	(0.106)	(0.139)	(0.168)
Observations	1541	786	755	1543	787	756
	***B. Working memory***					
	Working memory score			Score of 0		
Exogenous shock at birth	−0.107	0.143	−0.358	0.171	−0.038	0.456^+^
	(0.135)	(0.161)	(0.220)	(0.149)	(0.204)	(0.247)
Observations	1276	644	632	1276	644	632
	***C. Mathematical skills***					
	Math score			Score of 0		
Exogenous shock at birth	−0.790**	−0.493	−1.218**	0.239	−0.014	0.628**
	(0.249)	(0.350)	(0.367)	(0.152)	(0.206)	(0.239)
Observations	1510	770	740	1510	770	740
	***D. Schooling***					
	Educational attainment			Age for grade ⩾ 3		
Exogenous shock at birth	−0.103	0.055	−0.325*	0.095	0.021	0.244
	(0.101)	(0.132)	(0.159)	(0.113)	(0.150)	(0.178)
Observations	1554	792	762	1447	738	709

*Note*: Standard errors in parentheses clustered at the household level (^+^*p* < 0.10, *p < 0.05, **p < 0.01). All regressions control for age (in years) and region fixed effects, age and marital status of the caregiver at birth, educational level of the caregiver (no school, primary level of education, secondary level of education and higher of education), a continuous wealth index of the household and sex of the adolescent. “Exogenous shock at birth” is a dichotomous variable that takes the value 1 if the adolescent experienced a “poor crop yields” or a “big change in price of grain” economic shock at birth that affected other households in the community. ^*a*^: Lower values indicate worse cognition/education outcomes. ^*b*^: Higher values indicate worse cognition/education outcomes.

Overall, the associations appear to be more precisely estimated and similar in magnitude to those obtained in our benchmark analysis for adolescents who experienced two^+^ shocks at birth. Specifically, girls who experience an exogenous shock at birth have a lower weighted average derived from our continuous cognitive measures by about 0.272 points (Column 3, p-value = 0.010), whereas the association for boys is not statistically significant (Column 2, p-value = 0.887). The difference in these two associations is statistically significant at 5% (z-score = 2.099, p-value = 0.036). Results using the summary index derived from the set of discrete outcomes are in line with our benchmark results: girls who experience an exogenous shock at birth have a higher summary index (*β* = 0.415, p-value = 0.004), whereas this is not the case for boys (*β* = 0.034, p-value = 0.759). The difference in these associations between boys and girls is statistically significant (z-score = -2.102, p-value = 0.036). Similar patterns can be seen when breaking down the summary indices into their four components, where differences between girls and boys appear particularly marked in reading and mathematical skills.

## 6 Robustness checks

We detail in Appendix C the various tests that we have implemented to assess the robustness of our findings. Perhaps the two most significant departures from our benchmark estimations are the ones that explore economic shocks that could potentially be serially correlated and controlling for wealth score, a variable that is possibly endogenous. We briefly discuss these two issues below and refer to Appendix C for further details.

One of the concerns in our analysis could be that the associations estimated thus far are due to serially correlated shocks that happened prior or after the year of birth, and may not be the result of shocks happening during the year of birth. We show in Appendix C that this does not appear to be the case, as shocks occurring the year prior to the year of birth or two years after the year of birth do not lead to similar associations. Moreover, the associations between economic shocks at birth and lower cognitive scores are robust to controlling for the average number of shocks per year experienced by the household of the adolescent over the period 2003–2008.^15^ This underscores the importance of the long-term cognitive impact of shocks during the year of birth and supports the fact that the associations between economic shocks at birth and cognitive scores estimated thus far do indeed capture distress and shocks in the year of birth and not just heterogeneity in some latent and uncontrolled socioeconomic characteristics of the households.

Furthermore, our results appear to be robust to various measures of wealth that we use to control for the household’s socioeconomic background. One concern is that although the wealth score is constructed from durable household assets, it could also be an outcome variable, given that economic shocks could affect households’ wealth and become a pathway to impacting children’s cognitive outcomes. However, as detailed in Appendix C, we show that our results are robust to using various other socioeconomic status measures, including land ownership and wealth score measured in 2004, which predates most of the births of adolescents in our sample.

## 7 Possible mechanism

Our analysis thus far has established that economic shocks in the year of birth are negatively associated with girls’ cognitive and educational outcomes but such associations are not observed among boys. Because it is difficult to precisely establish when cognitive abilities are developed, a proper mediation analysis that investigates the pathways through which economic shocks experienced at birth affect cognitive outcomes among adolescents is difficult to implement with our data. Many (if not most) candidate mediators could be not only the determinants of cognitive abilities, but also the results of them. Nonetheless, in this section, we provide suggestive evidence of possible mechanisms that might explain our results.

One candidate is early-life physical development, as it is an important determinant of later-life cognitive outcomes. However, we discuss and show in Appendix D that adolescents who are subject to economic shocks at birth do not appear to differ in terms of height, either measured in 2017/2018 or in 2008, from those who do not experience any such shocks.

Another possible mechanism could be that households that experience economic shocks adopt more extreme gender attitudes, favoring investment in boys’ education over girls. In the face of adversity and tightening budget constraints, households may have to make difficult choices and may favor boys, or buffer boys from the consequences of shocks, over girls. The associations between economic shocks at birth and cognitive outcomes could therefore be moderated by gender attitudes and differences in educational investment. In Appendix D we provide suggestive evidence that supports this mechanism.

As discussed in Appendix D, we find that girls who experienced a negative economic shock during the year of birth received lower educational investment from their households compared to girls who did not experience any shocks at birth. We do not observe corresponding associations for boys. In turn, we show that investment in education at the household level predicts the cognitive and educational outcomes of the adolescents in our sample. We find that higher investment in education at the household level appears to be particularly beneficial for girls and less so for boys. This is consistent with the above results: girls’ outcomes are more sensitive to investment in education, and economic shocks decrease the amount of investment that is spent on their education.

Overall, our analyses of potential mechanisms suggests evidence that investment in education could be one of the reasons why we observe negative associations between economic shocks at birth and cognitive outcomes and educational attainment for girls but not for boys. We find evidence that these gender differences possibly and partially stem from changes in investment in education, where boys appear to be relatively protected from cuts in investment whereas girls suffer from investment cuts following negative economic shocks that occur during the year of their birth.

## 8 Discussion

Our study is among the first to examine the association of moderate, frequently-occurring shocks in early life on adolescent cognition and schooling attainment in a LIC. We find that two or more moderate economic shocks in the year of birth adversely affect adolescent girls’ educational and cognitive outcomes, though we do not observe the same pattern for boys, unlike previous studies (Beshir & Maystadt, 2020). We also find that effects on girls’ educational and cognitive outcomes are larger for shocks that affect the entire community, potentially making it difficult for households to buffer their impact by seeking help from neighbors. We also find suggestive evidence for educational investment as a possible pathway that might explain gender differences in the long-term impact of shocks. While we cannot formally test educational investment as a mediating mechanism, our results indicate that households compensate boys’ but not girls’ education in response to shocks in the year of birth. This is consistent with our expectation that lower educational investment in early childhood may be a possible pathway to girls’ disadvantage in educational and cognitive outcomes during adolescence.

As a possible biological pathway (based on limited sample size), we find no evidence that height mediate the relationship between shocks in the year of birth and adolescent educational outcomes. However, this measure may be too crude for capturing the cognitive impact of experiencing economic shocks in utero. Notably, we do not find that shocks experienced two years after birth affect either adolescent girls’ or boys’ educational and cognitive outcomes. Given that more recent shocks to household resources are expected to affect educational investments in children (Hyder et al. 2015), this finding hints at the possibility of biological mechanisms driving long-term gender differences in educational outcomes.

Overall, similar to evidence on pathways from Ethiopia (Beshir & Maystadt, 2020), our study encourages future investigation of both biological and social pathways that might help explain why in utero or early life shocks result in gender differences in adolescent’s educational and cognitive outcomes in low income countries. Our findings lend support to policies aimed at alleviating educational inequalities in Malawi (Psaki et al. 2018), and sub-Saharan Africa more broadly. Although the gender gap in primary school completion rates in Malawi has narrowed in recent years (Brossard et al. 2010; Psaki et al. 2018), overall primary school completion remain low. Despite seeming gender equality in low educational attainment among all adolescents, the pathways to school dropout may still be gendered. For instance, girls may experience drop out (and thus have low educational attainment) due to pregnancy, whereas boys may dropout of school to participate in paid work (Psaki et al. 2018). Differential pathways to school dropout require different interventions. Our results also highlight that economic shocks in the year of birth may be an additional gendered pathway that puts girls at an educational disadvantage. Therefore, policymakers should intervene early to alleviate the long-term educational impact of these shocks for girls. Refining the nature and design of such interventions may hinge on further evidence on what role biological and social pathways play in generating gender differences in educational outcomes. Evidence of detrimental impact on cognitive development in utero may imply greater investment in the health and well being of pregnant mothers, whereas reduced educational investment may suggest a need for early economic incentives for girls’ education. However, regardless of which mechanism is more dominant, existing social protection programs, such as cash transfer programs, could be used to assist households that experience multiple, negative shocks, particularly those with pregnant women.

The importance of our findings notwithstanding, our study has some limitations. First, for better causal interpretation, testing models with family fixed effects using sibling data would be useful, but we are unable to do so given data limitations. Second, household shocks in our study are self-reported and these reports may be subject to recall bias (this concern, however, is somewhat alleviated as shocks were reported by parents in 2008 and 2010 at the time when the adolescents were born, rather than being recalled retrospectively from more than a decade ago). Third, variation in cognitive scores based on age and grade level may yield a more nuanced understanding of the cognitive impact of shocks on the ability to learn progressively difficult concepts. Fourth, selective survival could potentially explain the difference in the associations we find across sex in the case where a higher fraction of male fetuses that were exposed to economic shocks die compared to female fetuses. The dataset at hand unfortunately does not allow to directly test this hypothesis. Following Currie et al. (2018), we can however perform an indirect test by using the sex of the adolescent at birth as a signal of changes to miscarriage rates, since male fetuses have a higher risk of miscarriage (Halla & Zweimüller,^2014^; Sanders & Stoecker, 2015). As reported in Appendix Table D9, experiencing economic shocks at birth does not predict the sex of the adolescents we have in our sample. This suggests that differential selection into birth because of miscarriages is unlikely to bias our results. Fifth, selective fertility could also be a threat to identification. Mothers who give birth in the year when an economic shock occurs might be different from those who decide to postpone their pregnancy. While we do control for household socioeconomic characteristics and caregiver’s education in our econometric specification, we cannot rule out the possible influence of selective fertility on the associations we estimate in this study.

Overall, our study is among the first to show evidence of girls’ long-term educational disadvantage as a result of experiencing multiple, moderate early life economic shocks. These shocks represent an additional pathway through which girls’ educational progress may be curtailed in Malawi. More broadly, our findings emphasize that LIC program developers and policymakers consider vulnerability from early life shocks as an important target for intervention, including early-life shocks that are “only” fairly commonly experienced in utero or during early life.

## Funding statement

Research reported in this publication was supported by the Eunice Kennedy Shriver National Institute of Child Health & Human Development of the 10.13039/100000002National Institutes of Health under Award Numbers R01HD090988, R01HD087391 and R01 RHD053781. The content is solely the responsibility of the authors and does not necessarily represent the official views of the National Institutes of Health. We also gratefully acknowledge the generous support from the Swiss Programme for Research on Global Issues for Development (SNF r4d Grant 400640_160374) and the 10.13039/501100001711Swiss National Science Foundation (grant number: P2LAP1_187736).

## Data availability statement

The public-use data of the Malawi Longitudinal Study for Families and Health (MLSFH) are available from https://malawi.pop.upenn.edu/malawi-data-mlsfh. Additional data of the MLSFH Adverse Childhood Experiences (ACE) project that are used for our analyses can be requested from the authors, and will ultimately be made publicly available as part of the MLSFH.

## Ethical statement

Ethics approvals were obtained both in the US and in Malawi. The authors have no relevant financial or non-financial interests to disclose.

## Declaration of competing interest

The authors have no relevant financial or non-financial interests to disclose.

## Footnotes

1 The economic shocks we use in our study are much more frequent: individuals who participated in the MLSFH survey in 2008 reported to have experienced on average 2.1 economic shocks between 2003 and 2008. 5, 22, 33 and 40% of the respondents reported 0,1,2 and 3 negative shocks, respectively.

2 Relative to other districts in Malawi, these districts fall in the middle and are not the most disadvantaged (Malawi Census Report 2018). Relative to other LICs in SSA, Malawi has a lower per capita income, but is similar in terms of life expectancy, infant mortality, child malnutrition, access to clean water, literacy, and schooling enrollment (World Bank, 2021).

3 Given how the MLSFH sample was selected, we expect adolescents in our sample to closely match the broader population of rural adolescents in Malawi.

4 These adolescents were selected from the 2008 and 2010 Household Rosters of the MLSFH.

5 Parents, grandparents and aunts/uncles represent 77%, 16% 3.4% of these individuals, respectively.

6 The 2008 questionnaire asked about shocks that occurred in the past five years, from 2003 to 2008, and the 2010 questionnaire asked about shocks that happened over the past two years, covering the period from 2008 to 2010.

7 Note that all adolescents in our sample were born between 2003 and 2008. We use shocks reported in the 2010 survey for the year 2008 so that we include economic shocks that occurred after the 2008 study.

8 “Age for grade” is a measure of delayed entry and/or grade repetition based on the adolescents’ age and the grade in which they should be as per the expected school schedule.

9 The summary index is a weighted mean of multiple standardized outcomes in which the weights are calculated through GLS weighting procedure to extract the maximum information from the various items. Missing items are ignored when creating the index. We do however control for the number of outcomes we observe per adolescent using dichotomous variables. Note also that we use those who did not experience any shock at birth as reference when normalizing the outcome variables. Our results are robust to using the entire sample as reference.

10 Loss of coupon pertains to loss of fertilizer coupon, which is an important factor of production for many individuals in rural Malawi.

11 Note that adolescents can experience up to three economic shocks at birth, but given the very low occurrence of experiencing three shocks –only 10 adolescents experienced three economic shocks at birth (0.64% of our sample)– we combine those who experienced three shocks with those who experienced two economic shocks at birth.

12 Because the wealth index can potentially be directly related to the (previous) experience of economic shocks, as a robustness check we use values of this variables prior to adolescent birth instead of the “current one”. Despite a notable decrease in the sample size, we show that results are qualitatively similar to those estimated in the specification with the full sample. More details are provided in the “Robustness checks” section.

13 Remember that the higher the summary index based on discrete outcomes is, the lower the cognitive outcomes.

14 Note that the last three columns in these panels report Probit coefficients, from which only the direction of the associations between economic shocks and the various cognitive outcomes can be straightforwardly inferred.

15 We exclude the shocks reported during the year of birth when computing the average number of shocks experienced by the households between 2003 and 2008.
