# Supplementary material for: The effects of negative economic shocks at birth on adolescents’ cognitive outcomes and educational attainment in Malawi
Source: SSM Popul Health. 2022 Apr 7;18:101085. doi: 10.1016/j.ssmph.2022.101085 (PMC9048075; doi:10.1016/j.ssmph.2022.101085)
Supplement: Multimedia component 1 [file mmc1.pdf]

Online Appendix

A Cognitive measures

The MLSFH ACE data provide several measures of cognitive abilities that encompass three different domains: literacy, mathematical skills, and working memory.

Reading score: Our initial outcome variable is "reading score", which ranges from 0 to 8. Adolescents were presented with four different sentences, two in Chichewa and two in English, that they were asked to read. For each of these sentences, they received a score of 0 if they were not able to read any part of the sentence, 1 point if they were able to read only part of the sentence, and 2 points if they were able to read the whole sentence. For our measure, we added scores for all four sentences. To explore the effects of economic shocks at birth at the lower end of the reading score distribution, we also created a dichotomous variable that takes the value 1 if adolescents were not able to read any part of the two Chichewa sentences, and 0 otherwise.

Mathematical skills: We created a measure for mathematical skills based on 12 questions that assess different analytical abilities.Therefore, the Math score ranges from 0 to 12. The first two questions asked adolescents to fill in missing numbers in a short sequence of numbers, and order five numbers in descending order. The next eight questions tested adolescents' ability to perform simple additions, subtractions, multiplications and divisions (two questions for each domain). The last two questions were two short word problems that adolescents were asked to solve. As an example, one of the word problems asked, "You have 75 tambala and you want to buy a pencil that cost 67 tambala. How much change would you get?". Adolescents were given one point for correctly answering each question. For our measure, we added their scores on each question. Similar to our measure for reading ability, we also created a dichotomous variable for Math that takes the value 1 if adolescents were not able to answer to any of the math questions correctly, and 0 otherwise.

Working memory: Our third cognitive measure is working memory. Adolescents were asked to repeat a list of numbers backwards. We created a measure of working memory that ranges from 0 to 7. A series of only two numbers was asked first, and became progressively larger, reaching a list of eight digits.[1] An adolescent is given a point for each additional number he or she is able to recall. A score of 0 is therefore given to an adolescent who is not able to repeat a list of two numbers and a score of 7 to someone who is able to repeat the list of eight numbers. As we did for our two previous outcome variables, we created a dichotomous variable for working memory that takes the value 1 if adolescents had a working memory score of 0, and 0 otherwise.

[1]This measure was added to the survey between rounds of data collection, and thus is only available for those respondents in the 2018 round.

B Additional descriptive statistics and details on economic shocks

Table B1: Number and type of shocks experienced by boys ($N = 794$) and girls ($N = 765$)

	Boys	Girls	Difference (p-value)
Number of shock			
0 shock at birth	0.739	0.766	0.222
1 shock at birth	0.209	0.178	0.118
2 shocks or more at birth	0.052	0.056	0.690
Type of shocks			
Death or serious illness	0.067	0.052	0.228
Poor crop yields	0.096	0.108	0.405
Loss income	0.042	0.039	0.814
Big change in price of grain	0.086	0.063	0.085
Fertilizer subsidy	0.001	0.001	0.979
Breakup of houshold	0.011	0.020	0.185
Damage to house	0.016	0.008	0.125
Other	0.001	0.001	0.979

Note: These shocks are reported by adolescents' households as part of the MLSFH collected in 2008 and 2010. P-values are the results of t-tests that compare the mean number of shocks experienced by adolescents in our sample by sex.

Table B2: Predictors of the number of shocks at birth

	Shock(s) at birth	Shock(s) at birth	Shock(s) at birth	Shock(s) at birth	Shock(s) at birth	Shock(s) at birth
Girl	-0.062	-0.062	-0.063	-0.015	0.227	0.237
	(0.068)	(0.068)	(0.068)	(0.123)	(0.312)	(0.363)
South region	0.040	0.042	0.056	0.158	0.165	0.190^{+}
	(0.079)	(0.079)	(0.078)	(0.107)	(0.107)	(0.109)
North region	0.027	0.025	-0.021	-0.054	-0.060	-0.090
	(0.082)	(0.082)	(0.092)	(0.114)	(0.114)	(0.126)
Caregiver married at birth		-0.027	-0.042		0.040	0.019
		(0.109)	(0.110)		(0.144)	(0.144)
Age of the caregiver at birth		0.003	0.004		0.005	0.006
		(0.003)	(0.003)		(0.003)	(0.004)
Primary level education - caregiver			0.055			0.091
			(0.088)			(0.122)
Secondary level education - caregiver			0.057			-0.079
			(0.147)			(0.209)
Wealth score			0.020			0.023
			(0.020)			(0.027)
Girl × South region				-0.257	-0.266	-0.283^{+}
				(0.167)	(0.167)	(0.165)
Girl × North region				0.148	0.148	0.122
				(0.171)	(0.171)	(0.190)
Girl × Caregiver married at birth					-0.125	-0.114
					(0.217)	(0.217)
Girl × Age of the caregiver at birth					-0.004	-0.004
					(0.006)	(0.006)
Girl × Primary level education - caregiver						-0.054
						(0.180)
Girl × Secondary level education - caregiver						0.269
						(0.300)
Girl × Wealth score						-0.013
						(0.039)
Observations	1559	1558	1556	1559	1558	1556
Test of joint-significance						
Wald $\chi^2(.)$-statistics	1.00	2.83	4.19	7.27	10.72	13.43
P-value	0.801	0.727	0.840	0.201	0.295	0.569

Note: Standard errors in parentheses clustered at the household level ($^{+}$ $p < 0.10$, * $p < 0.05$, ** $p < 0.01$). The coefficients are the results of ordered probit models for which the outcome variable is a categorical that takes the values 0, 1 or 2 for zero, one or two or more shocks experienced at birth, respectively. The sample is derived from the ACE sample collected in 2017 and 2018. Economic shocks are reported by adolescent's household as part of the MLSFH collected in 2008 and 2010.

Table B3: Predictors of experiencing poor crop yields at birth

	Poor crop yields	Poor crop yields	Poor crop yields	Poor crop yields	Poor crop yields	Poor crop yields
Girl	0.012	0.012	0.013	0.028	0.044	0.058
	(0.016)	(0.016)	(0.016)	(0.027)	(0.067)	(0.080)
South region	0.010	0.011	0.012	0.035	0.035	0.041
	(0.018)	(0.018)	(0.018)	(0.025)	(0.025)	(0.026)
North region	0.009	0.009	0.012	0.004	0.003	0.003
	(0.019)	(0.019)	(0.021)	(0.025)	(0.025)	(0.029)
Caregiver married at birth		0.007	0.009		0.006	0.006
		(0.023)	(0.023)		(0.032)	(0.032)
Age of the caregiver at birth		0.000	0.001		0.001	0.001
		(0.001)	(0.001)		(0.001)	(0.001)
Primary level education - caregiver			0.011			0.025
			(0.019)			(0.028)
Secondary level education - caregiver			0.015			0.010
			(0.035)			(0.048)
Wealth score			-0.004			-0.003
			(0.004)			(0.006)
Girl × South region				-0.050	-0.050	-0.057
				(0.038)	(0.038)	(0.038)
Girl × North region				0.009	0.010	0.016
				(0.039)	(0.039)	(0.043)
Girl × Caregiver married at birth					0.003	0.007
					(0.046)	(0.047)
Girl × Age of the caregiver at birth					-0.001	-0.001
					(0.001)	(0.001)
Girl × Primary level education - caregiver						-0.024
						(0.040)
Girl × Secondary level education - caregiver						0.011
						(0.073)
Girl × Wealth score						-0.003
						(0.008)
Observations	1559	1558	1556	1559	1558	1556
Test of joint-significance						
F-statistics	0.39	0.37	0.43	0.81	0.59	0.56
P-value	0.763	0.872	0.905	0.541	0.807	0.906

Note: Standard errors in parentheses clustered at the household level ($^{+}$ $p < 0.10$, * $p < 0.05$, ** $p < 0.01$). The coefficients are the results of linear regressions for which the outcome variable is a dichotomous variable that takes the values 1 if the adolescent experienced a "poor crop yields" shock at birth, and 0 otherwise. The sample is derived from the ACE sample collected in 2017 and 2018. Economic shocks are reported by adolescent's household as part of the MLSFH collected in 2008 and 2010.

Table B4: Predictors of experiencing big changes in the price of grain at birth

	Big change in price of grain	Big change in price of grain	Big change in price of grain	Big change in price of grain	Big change in price of grain	Big change in price of grain
Girl	-0.023^{+}	-0.023^{+}	-0.024^{+}	-0.031	-0.049	-0.040
	(0.013)	(0.013)	(0.013)	(0.023)	(0.059)	(0.067)
South region	0.017	0.017	0.021	0.018	0.018	0.025
	(0.016)	(0.016)	(0.016)	(0.024)	(0.024)	(0.025)
North region	0.005	0.005	0.003	-0.008	-0.008	-0.009
	(0.016)	(0.016)	(0.018)	(0.024)	(0.024)	(0.027)
Caregiver married at birth		0.008	0.007		0.000	-0.003
		(0.021)	(0.021)		(0.031)	(0.031)
Age of the caregiver at birth		0.000	0.000		0.000	0.000
		(0.001)	(0.001)		(0.001)	(0.001)
Primary level education - caregiver			0.020			0.031
			(0.017)			(0.027)
Secondary level education - caregiver			0.006			-0.013
			(0.034)			(0.041)
Wealth score			-0.000			-0.000
			(0.004)			(0.006)
Girl × South region				-0.002	-0.001	-0.007
				(0.032)	(0.032)	(0.032)
Girl × North region				0.027	0.028	0.024
				(0.032)	(0.032)	(0.036)
Girl × Caregiver married at birth					0.016	0.021
					(0.040)	(0.041)
Girl × Age of the caregiver at birth					0.000	0.000
					(0.001)	(0.001)
Girl × Primary level education - caregiver						-0.021
						(0.034)
Girl × Secondary level education - caregiver						0.042
						(0.053)
Girl × Wealth score						-0.001
						(0.008)
Observations	1559	1558	1556	1559	1558	1556
Test of joint-significance						
F-statistics	1.35	0.90	0.82	1.01	0.64	0.71
P-value	0.257	0.483	0.587	0.411	0.767	0.779

Note: Standard errors in parentheses clustered at the household level ($^{+}$ $p < 0.10$, * $p < 0.05$, ** $p < 0.01$). The coefficients are the results of linear regressions for which the outcome variable is a dichotomous variable that takes the values 1 if the adolescent experienced a "big change in the price of grain" shock at birth, and 0 otherwise. The sample is derived from the ACE sample collected in 2017 and 2018. Economic shocks are reported by adolescent's household as part of the MLSFH collected in 2008 and 2010.

Table B5: Descriptive statistics of adolescents in our sample by sex

	Boys	Girls	Difference (p-value)
Control variables			
Age	12.742	12.923	0.015
Central region	0.324	0.285	0.097
South region	0.368	0.374	0.803
North region	0.309	0.341	0.169
Age of the caregiver at birth	31.980	31.988	0.990
Caregiver married at birth	0.870	0.873	0.863
No formal education - caregiver	0.273	0.251	0.317
Primary level education - caregiver	0.641	0.667	0.288
Secondary level education or higher - caregiver	0.086	0.082	0.815
Wealth score	-0.153	-0.005	0.118

Note: The sample is derived from the ACE sample collected in 2017 and 2018. Economic shocks are reported by adolescent's household as part of the MLSFH collected in 2008 and 2010. P-values are the results of t-tests that compare the mean characteristics of adolescents in our sample by sex.

C Robustness checks

We test other specifications to check the robustness of our findings. First, our benchmark results pertaining to the associations between negative economic shocks and our two summary indices use adolescents who do not experience any shock at birth as (sex-specific) reference group to normalize the outcomes. We show in Tables C1 and C2 that using a norm based on 1) those who did not experience any shock at birth irrespective of sex, 2) sex only irrespective of the number of shocks experienced at birth, or 3) the entire sample leads to very similar results.

Second, Table C3 presents the results obtained when regressing our set of discrete outcome variables on economic shocks at birth using OLS instead of Probit regressions. The resulting associations are in line with those presented in the main text.

Third, Tables C4 and C5 present corresponding analysis but this time interacting economic shocks during the year of birth with sex, instead of running separate regressions by sex. This new specification is not as flexible as our benchmark specification as it has the undesirable property to "force" coefficients—other than economic shocks—to be similar across sex. Because processes and characteristics that govern cognitive and educational outcomes can vary across gender in this context (UNICEF 2020), such a specification is probably too restrictive and unlikely to fit the data well. We allow not only shocks but also age effects to depend on sex, in an attempt to resolve a part of this restriction. Similar to our benchmark analysis, no clear relationship between the occurrence of economic shocks during the year of birth and cognitive outcomes can be observed for boys. A clear negative relationship can be seen for girls however, for whom the detrimental effects become larger as the number of economic shocks occurring during the year of birth increases.

Fourth, we present in Table C6 further results that reinforce the possibility of interpreting our associations as being causal. More specifically, we report the results from a specification in which we do not control for potentially endogenous independent variables (age of caregiver at birth, education level of caregiver, marital status of caregiver at birth and household wealth index). Panel A of that table shows that our benchmark estimates are robust to that specification, suggesting that our economic shocks are indeed exogenous. Moreover, Panel B presents results in which we include village fixed-effects instead of region fixed-effects in our analysis. This departure from our benchmark specification can better capture unobserved local characteristics that could potentially affect both the occurrence of economic shocks and cognitive function, therefore further strengthening the causal interpretation of our results. Panel B shows again that results are very similar to the ones reported in the main table. Finally, Panel C and D show corresponding results in which we add the number of children in the

household and birth order in the econometric specification. Once again, our results appear to be quantitatively similar to our benchmark results, despite the smaller sample size (Panel D).

One concern in our analysis is that the associations estimated thus far could be due to serially correlated shocks that happened prior or after the year of birth, and may not be the result of shocks happening during the year of birth. To rule out this possibility, we include in the same econometric specification both economic shocks occurring during the year of birth and those occurring two years after the year of birth.[2] Table C7 shows that including economic shocks two years after birth does not alter the associations between cognitive function and economic shocks occurring in the year at birth. Note also that including only economic shocks occurring two years after birth does not explain any of our outcome variables (Table C8). In the same spirit, economic shocks occurring the year prior to the year of birth are not associated with lower cognitive characteristics either (Table C9). Finally we show in Table C10 that the associations between economic shocks at birth and lower cognitive skills are robust to controlling for the average number of shocks per year experienced by the household of the adolescent over the period 2003-2008.[3] This underscores the importance of the long-term cognitive impact of shocks during the year of birth and supports the fact that the associations between economic shocks at birth and cognitive function estimated thus far does indeed capture distress and shocks in the year of birth and not just heterogeneity in some latent and uncontrolled socioeconomic characteristics of the households.

An additional concern is that the wealth score we include as a control variable in our benchmark specification could also be an outcome variable, given that economic shocks could affect households' wealth and become a pathway to impacting children's cognitive health and outcomes. Although our wealth measure is constructed from durable household assets that may be relatively stable over time, including such an independent variable in our model could capture some of the associations between economic shocks at birth cognitive and educational outcomes. However, we show that our results are robust to other versions of wealth measures. First, instead of a wealth score, we include in our model household land ownership as a proxy for socioeco-

[2]We include in the econometric specification economic shocks occurring two years after the year of birth to make sure these shocks do not happen right after birth, for instance for those born in December of a given year. Moreover, given that adolescents in our sample are born between 2003 and 2008 and that we have information about the occurrence of economic shocks covering the period from 2003 to 2010, we cannot investigate the associations between cognitive function and economic shocks that occur beyond two years after the year of birth for those born in 2008.

[3]We exclude the shocks reported during the year of birth when computing the average number of shocks experienced by the households between 2003 and 2008.

nomic status. Table C11 shows that controlling for land ownership does not alter our previous findings.[4] In addition, the only assets for which we have information about whether respondents owned them in 1998, 2001, 2004, 2006, 2008 and 2010 were mattress, radio, bicycle, pit latrine and lamp. Using this subset of assets, we created an indicator variable that takes the value 1 if a respondent owned a particular asset, and summed these indicator variables over the five different assets. We did that for survey years 1998, 2001, 2004, 2006, 2008 and 2010, and took the average of these asset scores, conditioning again on having at least two observations. Table C12 shows that our results are robust to including this measure of socioeconomic status. Table C13 also presents results controlling for wealth score measured in 2004, which predates most of the births of adolescents in our sample. Despite the large decrease in the sample size since not all adolescents had caregivers that were interviewed in 2004, Table C13 shows that the associations between economic shocks and cognitive and educational outcomes are similar to those estimated in our benchmark specification, but less precisely estimated due to smaller sample size. We also present results from a specification in which wealth score is not controlled for. In that case, under the assumption that economic shocks and wealth score are negatively correlated, we would expect economic shocks to have larger associations on cognitive and educational outcomes than when wealth score is controlled for.[5] This is indeed what we see in our results (Table C14). Overall, our results are robust to various specifications of wealth measures, including wealth measures that preceded the birth of the adolescents in our sample.

[4]To construct our measure of land ownership, we compute the percentile rank of respondent's land ownership in each wave 1998, 2001, 2004, 2006, 2008 and 2010, and take the average of these percentile ranks, conditioning on having at least two observations for each respondents.

[5]Not including wealth index in the analysis therefore helps to identify the *total associations* of the shocks on cognitive outcome, but weakens the plausibility of the exogeneity of the shocks.

Table C1: Associations between economic shock at birth and summary indices - continuous outcomes. Robustness checks based on the reference group used as norm

	All (1)	Boys (2)	Girls (3)
No shock at birth, irrespective of sex			
1 shock at birth	0.025	0.062	-0.032
	(0.056)	(0.076)	(0.084)
2 shocks or more at birth	-0.164	-0.011	-0.330*
	(0.103)	(0.131)	(0.153)
Observations	1554	792	762
Sex, irrespective of the number of shocks at birth			
1 shock at birth	0.026	0.060	-0.030
	(0.056)	(0.079)	(0.085)
2 shocks or more at birth	-0.169	-0.024	-0.334*
	(0.104)	(0.136)	(0.155)
Observations	1554	792	762
Entire sample			
1 shock at birth	0.026	0.060	-0.028
	(0.056)	(0.077)	(0.085)
2 shocks or more at birth	-0.169	-0.020	-0.332*
	(0.104)	(0.133)	(0.153)
Observations	1554	792	762

Note: The sample is derived from the ACE sample collected in 2017 and 2018. Economic shocks are reported by adolescent's household as part of the MLSFH collected in 2008 and 2010. All regressions control for age (in years) and region fixed effects, age and marital status of the caregiver at birth, educational level of the caregiver (no school, primary level of education, secondary level of education and higher of education), a continuous wealth index of the household and sex of the adolescent. Standard errors are clustered at the household level ($^{+}$ $p < 0.10$, * $p < 0.05$, ** $p < 0.01$). The summary index is a standardized weighted index based on our four continuous variables following a GLS weighting procedure as described in (Anderson 2008).

Table C2: Associations between economic shock at birth and summary indices - discrete outcomes. Robustness checks based on the reference group used as norm

	All<br>(1)	Boys<br>(2)	Girls<br>(3)
No shock at birth, irrespective of sex			
1 shock at birth	0.030	-0.022	0.088
	(0.067)	(0.095)	(0.101)
2 shocks or more at birth	0.302*	0.090	0.517*
	(0.142)	(0.187)	(0.207)
Observations	1554	792	762
Sex, irrespective of the number of shocks at birth			
1 shock at birth	0.031	-0.017	0.086
	(0.063)	(0.086)	(0.098)
2 shocks or more at birth	0.282*	0.077	0.514*
	(0.137)	(0.177)	(0.207)
Observations	1554	792	762
Entire sample			
1 shock at birth	0.031	-0.017	0.085
	(0.063)	(0.088)	(0.094)
2 shocks or more at birth	0.282*	0.077	0.491*
	(0.137)	(0.180)	(0.220)
Observations	1554	792	762

Note: The sample is derived from the ACE sample collected in 2017 and 2018. Economic shocks are reported by adolescent's household as part of the MLSFH collected in 2008 and 2010. All regressions control for age (in years) and region fixed effects, age and marital status of the caregiver at birth, educational level of the caregiver (no school, primary level of education, secondary level of education and higher of education), a continuous wealth index of the household and sex of the adolescent. Standard errors are clustered at the household level ($^{+}$ $p < 0.10$, * $p < 0.05$, ** $p < 0.01$). The summary index is a standardized weighted index based on our four continuous variables following a GLS weighting procedure as described in (Anderson 2008).

Table C3: Associations between economic shock at birth and discrete measures of cognitive health, using OLS instead of Probit

	All (1)	Boys (2)	Girls (3)
	A. Reading skills		
	Can't read Chichewa		
1 shock at birth	-0.009	-0.014	0.006
	(0.029)	(0.042)	(0.041)
2 shocks or more at birth	0.097^{+}	0.057	0.144^{+}
	(0.055)	(0.079)	(0.077)
Observations	1543	787	756
	B. Working memory		
	Score of 0		
1 shock at birth	0.000	-0.038	0.049
	(0.019)	(0.025)	(0.031)
2 shocks or more at birth	0.093^{+}	0.076	0.112^{+}
	(0.048)	(0.072)	(0.065)
Observations	1276	644	632
	C. Mathematical skills		
	Score of 0		
1 shock at birth	0.015	0.005	0.027
	(0.018)	(0.026)	(0.024)
2 shocks or more at birth	0.034	-0.029	0.095^{+}
	(0.037)	(0.051)	(0.054)
Observations	1510	770	740
	D. Schooling		
	Age for grade $\geqslant 3$		
1 shock at birth	-0.006	-0.012	0.004
	(0.029)	(0.039)	(0.044)
2 shocks or more at birth	0.045	0.012	0.088
	(0.055)	(0.081)	(0.078)
Observations	1447	738	709

Note: The sample is derived from the ACE sample collected in 2017 and 2018. Economic shocks are reported by adolescent's household as part of the MLSFH collected in 2008 and 2010. All regressions control for age (in years) and region fixed effects, age and marital status of the caregiver at birth, educational level of the caregiver (no school, primary level of education, secondary level of education and higher of education), a continuous wealth index of the household and sex of the adolescent. Standard errors are clustered at the household level ($^{+}$ $p < 0.10$, * $p < 0.05$, ** $p < 0.01$). These coefficients are the results of OLS regressions.

Table C4: Associations between economic shocks at birth and continuous outcome variables - interactions between shock and sex

Total interactions	Summary index continuous (1)	Reading score (2)	Working memory (3)	Math score (4)	Educational attainment (5)
Sex-specific age dummies					
1 shock at birth - Boys	0.066	0.234	0.101	-0.003	0.109
	(0.075)	(0.287)	(0.148)	(0.298)	(0.113)
2 shocks or more at birth - Boys	0.002	0.045	-0.069	-0.418	0.208
	(0.130)	(0.489)	(0.243)	(0.524)	(0.202)
1 shock at birth - Girls	-0.028	-0.023	-0.077	-0.111	-0.101
	(0.083)	(0.299)	(0.181)	(0.319)	(0.133)
2 shocks or more at birth - Girls	-0.330*	-0.926^{+}	-0.527^{+}	-0.739	-0.415^{+}
	(0.151)	(0.526)	(0.311)	(0.540)	(0.242)
Observations	1554	1541	1276	1510	1554

Note: The sample is derived from the ACE sample collected in 2017 and 2018. Economic shocks are reported by adolescent's household as part of the MLSFH collected in 2008 and 2010. All regressions control for age (in years) and region fixed effects, age and marital status of the caregiver at birth, educational level of the caregiver (no school, primary level of education, secondary level of education and higher of education), a continuous wealth index of the household and sex of the adolescent. Age dummy variables are also interacted with sex. Standard errors are clustered at the household level ($^{+}$ $p < 0.10$, * $p < 0.05$, ** $p < 0.01$).

Table C5: Associations between economic shocks at birth and discrete outcome variables - interactions between shock and sex

Total interactions	Index discrete (1)	Can't read Chichewa (2)	WM score of 0 (3)	Math score of 0 (4)	Age for grade $\geqslant 3$ (5)
Sex-specific age dummies					
1 shock at birth - Boys	-0.032	-0.041	-0.276	0.040	-0.015
	(0.091)	(0.125)	(0.218)	(0.173)	(0.130)
2 shocks or more at birth - Boys	0.060	0.140	0.282	-0.186	-0.014
	(0.186)	(0.235)	(0.308)	(0.312)	(0.252)
1 shock at birth - Girls	0.103	0.023	0.325	0.321	-0.023
	(0.097)	(0.145)	(0.205)	(0.219)	(0.142)
2 shocks or more at birth - Girls	0.538*	0.433^{+}	0.701*	0.696*	0.238
	(0.212)	(0.227)	(0.319)	(0.294)	(0.252)
Observations	1554	1543	1276	1510	1447

Note: The sample is derived from the ACE sample collected in 2017 and 2018. Economic shocks are reported by adolescent's household as part of the MLSFH collected in 2008 and 2010. All regressions control for age (in years) and region fixed effects, age and marital status of the caregiver at birth, educational level of the caregiver (no school, primary level of education, secondary level of education and higher of education), a continuous wealth index of the household and sex of the adolescent. Age dummy variables are also interacted with sex. Standard errors are clustered at the household level ($^{+}$ $p < 0.10$, * $p < 0.05$, ** $p < 0.01$).

Table C6: Further robustness checks on the associations between economic shocks at birth and our cognitive summary indices

	All (1)	Boys (2)	Girls (3)	All (4)	Boys (5)	Girls (6)
	Summary index					
	Continuous outcomes			Discrete outcomes		
	A. Controlling for sex and age only					
1 shock at birth	0.041	0.075	-0.005	0.027	-0.025	0.086
	(0.062)	(0.081)	(0.096)	(0.071)	(0.093)	(0.113)
2 shocks at birth	-0.196^{+}	-0.097	-0.314^{+}	0.350*	0.161	0.574*
	(0.111)	(0.134)	(0.174)	(0.155)	(0.182)	(0.250)
Observations	1557	793	764	1557	793	764
	B. Village fixed-effects					
1 shock at birth	0.003	0.013	-0.012	0.048	0.044	0.048
	(0.057)	(0.086)	(0.089)	(0.067)	(0.096)	(0.117)
2 shocks at birth	-0.143	-0.054	-0.378*	0.258^{+}	0.124	0.521*
	(0.104)	(0.155)	(0.158)	(0.140)	(0.188)	(0.227)
Observations	1554	792	762	1554	792	762
	C. Controlling for # of children					
1 shock at birth	0.025	0.062	-0.035	0.030	-0.019	0.090
	(0.056)	(0.077)	(0.085)	(0.066)	(0.087)	(0.103)
2 shocks at birth	-0.160	-0.012	-0.332*	0.302*	0.093	0.549*
	(0.103)	(0.132)	(0.155)	(0.143)	(0.181)	(0.217)
Observations	1554	792	762	1554	792	762
	D. Controlling for # of children and birth order					
1 shock at birth	0.002	0.014	-0.078	0.049	0.002	0.160
	(0.072)	(0.105)	(0.108)	(0.090)	(0.117)	(0.139)
2 shocks at birth	0.086	0.244	-0.231	0.014	-0.131	0.380
	(0.134)	(0.175)	(0.185)	(0.189)	(0.229)	(0.346)
Observations	879	439	440	879	439	440

Note: The sample is derived from the ACE sample collected in 2017 and 2018. Economic shocks are reported by adolescent's household as part of the MLSFH collected in 2008 and 2010. Standard errors are clustered at the household level ($^{+}$ $p < 0.10$, * $p < 0.05$, ** $p < 0.01$). Panel A presents results when we control only for sex and age in the econometric specification. Panel B shows results when we use village fixed-effects instead of region fixed-effects. Panel C adds the number of children in the household as control variable. Panel D shows results in which both the number of children in the household and birth order are controlled for.

Table C7: Associations between economic shocks at birth and cognitive outcomes and educational attainment - including also shocks occurring two years after the year of birth

	All (1)	Boys (2)	Girls (3)	All (4)	Boys (5)	Girls (6)
	Summary index					
	Continuous outcomes			Discrete outcomes		
1 shock at birth	0.050	0.086	0.000	0.014	-0.047	0.084
	(0.058)	(0.081)	(0.088)	(0.068)	(0.091)	(0.108)
2 shocks or more at birth	-0.173	0.016	-0.398*	0.318*	0.077	0.585**
	(0.109)	(0.142)	(0.159)	(0.151)	(0.194)	(0.221)
1 shock 2 years after birth	0.014	0.025	0.012	-0.062	-0.041	-0.107
	(0.053)	(0.072)	(0.078)	(0.055)	(0.075)	(0.082)
2 shocks or more 2 years after birth	0.119^{+}	0.094	0.159^{+}	-0.056	-0.074	-0.012
	(0.070)	(0.097)	(0.097)	(0.084)	(0.111)	(0.116)
	A. Reading skills					
	Reading score			Can't read Chichewa		
1 shock at birth	0.180	0.245	0.054	-0.030	-0.051	-0.002
	(0.211)	(0.300)	(0.306)	(0.096)	(0.129)	(0.154)
2 shocks or more at birth	-0.486	-0.033	-0.954^{+}	0.330^{+}	0.194	0.506*
	(0.386)	(0.532)	(0.547)	(0.177)	(0.253)	(0.249)
1 shock 2 years after birth	0.054	0.058	0.078	-0.085	-0.108	-0.074
	(0.179)	(0.255)	(0.249)	(0.086)	(0.112)	(0.132)
2 shocks or more 2 years after birth	0.358	0.208	0.527	-0.062	0.048	-0.223
	(0.252)	(0.356)	(0.348)	(0.113)	(0.153)	(0.167)
	B. Working memory					
	Working memory score			Score of 0		
1 shock at birth	0.094	0.189	0.009	-0.062	-0.439^{+}	0.312
	(0.119)	(0.158)	(0.187)	(0.153)	(0.240)	(0.232)
2 shocks or more at birth	-0.298	-0.066	-0.599^{+}	0.478*	0.421	0.609^{+}
	(0.223)	(0.265)	(0.340)	(0.232)	(0.319)	(0.368)
1 shock 2 years after birth	0.082	0.146	0.012	-0.113	0.004	-0.316
	(0.115)	(0.151)	(0.171)	(0.129)	(0.172)	(0.212)
2 shocks or more 2 years after birth	0.204	0.189	0.272	-0.126	-0.276	0.006
	(0.151)	(0.191)	(0.206)	(0.177)	(0.253)	(0.249)
	C. Math skills					
	Math score			Score of 0		
1 shock at birth	-0.045	-0.072	-0.025	0.155	0.055	0.334
	(0.221)	(0.310)	(0.325)	(0.140)	(0.177)	(0.235)
2 shocks or more at birth	-0.494	-0.522	-0.585	0.210	-0.204	0.898**
	(0.400)	(0.561)	(0.567)	(0.233)	(0.342)	(0.330)
1 shock 2 years after birth	-0.131	-0.275	0.084	-0.091	-0.121	-0.049
	(0.190)	(0.265)	(0.271)	(0.126)	(0.160)	(0.220)
2 shocks or more 2 years after birth	0.136	-0.148	0.439	-0.157	-0.183	-0.082
	(0.266)	(0.356)	(0.380)	(0.173)	(0.221)	(0.256)
	D. Schooling					
	Educational attainment			Age for grade $\geqslant$ 3		
1 shock at birth	0.019	0.124	-0.120	-0.028	-0.062	0.020
	(0.090)	(0.117)	(0.139)	(0.099)	(0.132)	(0.152)
2 shocks or more at birth	-0.109	0.295	-0.508*	0.047	-0.133	0.270
	(0.173)	(0.227)	(0.251)	(0.194)	(0.267)	(0.285)
1 shock 2 years after birth	0.002	-0.017	0.037	-0.054	-0.024	-0.106
	(0.086)	(0.115)	(0.124)	(0.087)	(0.125)	(0.127)
2 shocks or more 2 years after birth	0.057	0.062	0.058	0.115	0.030	0.178
	(0.109)	(0.152)	(0.154)	(0.121)	(0.166)	(0.171)

Note: The sample is derived from the ACE sample collected in 2017 and 2018. Economic shocks are reported by adolescent's household as part of the MLSFH collected in 2008 and 2010. All regressions control for age (in years) and region fixed effects, age and marital status of the caregiver at birth, educational level of the caregiver (no school, primary level of education, secondary level of education and higher of education), a continuous wealth index of the household and sex of the adolescent. Standard errors are clustered at the household level ($^{+}$ $p < 0.10$, * $p < 0.05$, ** $p < 0.01$).

Table C8: Associations between economic shocks at birth and cognitive outcomes and educational attainment - including only shocks occurring two years after the year of birth

	All (1)	Boys (2)	Girls (3)	All (1)	Boys (2)	Girls (3)
	Summary index					
	Continuous outcomes			Discrete outcomes		
1 shock 2 years after birth	0.015	0.022	0.023	-0.070	-0.041	-0.129
	(0.052)	(0.072)	(0.077)	(0.055)	(0.074)	(0.082)
2 shocks or more 2 years after birth	0.115^{+}	0.089	0.156	-0.061	-0.074	-0.023
	(0.070)	(0.096)	(0.095)	(0.082)	(0.108)	(0.114)
	A. Reading skills					
	Reading score			Can't read Chichewa		
1 shock 2 years after birth	0.054	0.050	0.099	-0.092	-0.111	-0.090
	(0.178)	(0.254)	(0.249)	(0.085)	(0.111)	(0.132)
2 shocks or more 2 years after birth	0.343	0.198	0.509	-0.062	0.046	-0.221
	(0.250)	(0.353)	(0.346)	(0.111)	(0.151)	(0.165)
	B. Working memory					
	Working memory score			Score of 0		
1 shock 2 years after birth	0.081	0.144	0.018	-0.118	-0.007	-0.365^{+}
	(0.114)	(0.150)	(0.171)	(0.128)	(0.170)	(0.212)
2 shocks or more 2 years after birth	0.198	0.183	0.255	-0.139	-0.300	-0.071
	(0.150)	(0.190)	(0.203)	(0.177)	(0.248)	(0.243)
	C. Math skills					
	Math score			Score of 0		
1 shock 2 years after birth	-0.116	-0.260	0.101	-0.106	-0.119	-0.120
	(0.189)	(0.265)	(0.269)	(0.126)	(0.159)	(0.216)
2 shocks or more 2 years after birth	0.147	-0.129	0.439	-0.167	-0.177	-0.155
	(0.264)	(0.354)	(0.374)	(0.172)	(0.222)	(0.251)
	D. Schooling					
	Educational attainment			Age for grade $\geqslant$ 3		
1 shock 2 years after birth	0.003	-0.028	0.060	-0.053	-0.018	-0.115
	(0.085)	(0.114)	(0.122)	(0.086)	(0.124)	(0.125)
2 shocks or more 2 years after birth	0.056	0.048	0.077	0.118	0.039	0.180
	(0.107)	(0.151)	(0.149)	(0.120)	(0.164)	(0.169)

Note: The sample is derived from the ACE sample collected in 2017 and 2018. Economic shocks are reported by adolescent's household as part of the MLSFH collected in 2008 and 2010. All regressions control for age (in years) and region fixed effects, age and marital status of the caregiver at birth, educational level of the caregiver (no school, primary level of education, secondary level of education and higher of education), a continuous wealth index of the household and sex of the adolescent. Standard errors are clustered at the household level ($^{+}$ $p < 0.10$, * $p < 0.05$, ** $p < 0.01$).

Table C9: Associations between economic shocks at birth and cognitive outcomes and educational attainment - including only shocks occurring one year before the year of birth

	All (1)	Boys (2)	Girls (3)	All (4)	Boys (5)	Girls (6)
	Summary index					
	Continuous outcomes			Discrete outcomes		
1 shock 1 year before birth	0.083	0.084	0.071	-0.011	-0.023	-0.027
	(0.072)	(0.097)	(0.115)	(0.086)	(0.118)	(0.123)
2 shocks or more 1 year before birth	0.041	0.146	-0.020	0.134	-0.124	0.335
	(0.152)	(0.181)	(0.266)	(0.284)	(0.263)	(0.493)
	A. Reading skills					
	Reading score			Can't read Chichewa		
1 shock 1 year before birth	-0.070	-0.023	-0.167	0.025	-0.078	0.214
	(0.251)	(0.322)	(0.396)	(0.115)	(0.149)	(0.178)
2 shocks or more 1 year before birth	-0.030	0.259	-0.409	0.070	0.017	0.186
	(0.610)	(0.670)	(1.035)	(0.302)	(0.406)	(0.454)
	B. Working memory					
	Working memory score			Score of 0		
1 shock 1 year before birth	0.169	0.040	0.276	0.226	0.212	0.260
	(0.156)	(0.212)	(0.242)	(0.163)	(0.218)	(0.276)
2 shocks or more 1 year before birth	0.017	0.098	0.082	-0.500	-[a]	-0.288
	(0.245)	(0.243)	(0.466)	(0.548)		(0.823)
	C. Math skills					
	Math score			Score of 0		
1 shock 1 year before birth	0.082	-0.010	0.229	0.035	-0.032	-0.041
	(0.273)	(0.359)	(0.405)	(0.155)	(0.194)	(0.271)
2 shocks or more 1 year before birth	-0.341	0.014	-0.531	0.420	0.174	0.283
	(0.595)	(0.867)	(0.911)	(0.353)	(0.627)	(0.477)
	D. Schooling					
	Educational attainment			Age for grade $\geqslant 3$		
1 shock 1 year before birth	0.108	0.182	0.012	-0.133	-0.052	-0.244
	(0.110)	(0.134)	(0.186)	(0.121)	(0.162)	(0.192)
2 shocks or more 1 year before birth	0.059	0.179	0.005	0.143	-0.166	0.299
	(0.248)	(0.281)	(0.430)	(0.320)	(0.436)	(0.447)

Note: The sample is derived from the ACE sample collected in 2017 and 2018. Economic shocks are reported by adolescent's household as part of the MLSFH collected in 2008 and 2010. All regressions control for age (in years) and region fixed effects, age and marital status of the caregiver at birth, educational level of the caregiver (no school, primary level of education, secondary level of education and higher of education), a continuous wealth index of the household and sex of the adolescent. Standard errors are clustered at the household level ($^{+}$ $p < 0.10$, * $p < 0.05$, ** $p < 0.01$). [a]: When looking at the probability of having a cognitive skills score of 0, no coefficient for boys who experience two or more economic shocks at birth can be estimated because of lack of variation.

Table C10: Associations between economic shocks at birth and cognitive and educational attainment outcomes, controlling for average number of shocks per year

	All (1)	Boys (2)	Girls (3)	All (4)	Boys (5)	Girls (6)
	Summary index					
	Continuous outcomes			Discrete outcomes		
1 shock at birth	0.041	0.083	-0.024	0.026	-0.009	0.057
	(0.059)	(0.081)	(0.090)	(0.069)	(0.091)	(0.108)
2 shocks or more at birth	-0.145	0.047	-0.349*	0.288+	0.026	0.544*
	(0.117)	(0.148)	(0.175)	(0.156)	(0.190)	(0.233)
	A. Reading skills					
	Reading score			Can't read Chichewa		
1 shock at birth	0.175	0.320	-0.045	-0.021	-0.040	0.027
	(0.213)	(0.306)	(0.308)	(0.097)	(0.131)	(0.155)
2 shocks or more at birth	-0.449	0.057	-0.973+	0.303	0.206	0.430
	(0.405)	(0.547)	(0.583)	(0.185)	(0.261)	(0.262)
	B. Working memory					
	Working memory score			Score of 0		
1 shock at birth	0.032	0.105	-0.040	0.019	-0.213	0.260
	(0.119)	(0.155)	(0.188)	(0.155)	(0.220)	(0.245)
2 shocks or more at birth	-0.279	-0.056	-0.507	0.491*	0.571+	0.557
	(0.227)	(0.275)	(0.340)	(0.245)	(0.342)	(0.373)
	C. Mathematical skills					
	Math score			Score of 0		
1 shock at birth	-0.018	-0.011	-0.088	0.209	0.145	0.341
	(0.229)	(0.321)	(0.335)	(0.143)	(0.175)	(0.250)
2 shocks or more at birth	-0.582	-0.302	-0.999+	0.276	-0.284	0.863*
	(0.422)	(0.585)	(0.575)	(0.250)	(0.404)	(0.344)
	D. Schooling					
	Educational attainment			Age for grade $\geqslant 3$		
1 shock at birth	0.007	0.122	-0.148	-0.052	-0.081	-0.022
	(0.094)	(0.122)	(0.144)	(0.101)	(0.137)	(0.152)
2 shocks or more at birth	-0.108	0.280	-0.465	0.063	-0.113	0.266
	(0.190)	(0.235)	(0.286)	(0.202)	(0.291)	(0.287)

Note: Standard errors in parentheses clustered at the household level ($^{+}$ $p < 0.10$, * $p < 0.05$, ** $p < 0.01$). The sample is derived from the ACE sample collected in 2017 and 2018. Economic shocks are reported by adolescent's households as part of the MLSFH collected in 2008 and 2010. All regressions control for age (in years) and region fixed effects, age and marital status of the caregiver at birth, educational level of the caregiver (no school, primary level of education, secondary level of education and higher of education), a continuous wealth index of the household and sex of the adolescent.

Table C11: Associations between economic shocks at birth and cognitive outcomes and educational attainment - land ownership (percentile rank)

	All (1)	Boys (2)	Girls (3)	All (4)	Boys (5)	Girls (6)
	Summary index					
	Continuous outcomes			Discrete outcomes		
1 shock at birth	0.039	0.106	-0.054	0.022	-0.051	0.107
	(0.059)	(0.080)	(0.090)	(0.068)	(0.091)	(0.109)
2 shocks or more at birth	-0.145	-0.022	-0.315^{+}	0.317*	0.115	0.585*
	(0.111)	(0.140)	(0.172)	(0.152)	(0.187)	(0.240)
Observations	1463	737	726	1463	737	726
	A. Reading skills					
	Reading score			Can't read Chichewa		
1 shock at birth	0.175	0.332	-0.068	-0.031	-0.067	0.052
	(0.214)	(0.304)	(0.309)	(0.095)	(0.128)	(0.151)
2 shocks or more at birth	-0.379	-0.069	-0.809	0.263	0.205	0.394
	(0.376)	(0.518)	(0.559)	(0.167)	(0.239)	(0.248)
Observations	1450	731	719	1452	732	720
	B. Working memory					
	Working memory score			Score of 0		
1 shock at birth	0.047	0.212	-0.156	0.011	-0.393^{+}	0.489*
	(0.120)	(0.154)	(0.191)	(0.152)	(0.236)	(0.230)
2 shocks or more at birth	-0.286	-0.141	-0.473	0.502*	0.392	0.745*
	(0.216)	(0.261)	(0.330)	(0.225)	(0.314)	(0.358)
Observations	1202	600	602	1202	600	518
	C. Math skills					
	Math score			Score of 0		
1 shock at birth	-0.005	0.095	-0.168	0.163	0.050	0.381
	(0.223)	(0.313)	(0.332)	(0.141)	(0.184)	(0.232)
2 shocks or more at birth	-0.554	-0.430	-0.816	0.242	-0.092	0.850**
	(0.404)	(0.549)	(0.590)	(0.220)	(0.319)	(0.326)
Observations	1421	716	705	1421	716	648
	D. Schooling					
	Educational attainment			Age for grade $\geqslant 3$		
1 shock at birth	0.028	0.136	-0.139	-0.045	-0.049	-0.022
	(0.090)	(0.115)	(0.141)	(0.099)	(0.132)	(0.150)
2 shocks or more at birth	-0.080	0.209	-0.442	0.108	0.018	0.275
	(0.175)	(0.220)	(0.276)	(0.184)	(0.267)	(0.273)
Observations	1463	737	726	1366	689	677

Note: The sample is derived from the ACE sample collected in 2017 and 2018. Economic shocks are reported by adolescent's household as part of the MLSFH collected in 2008 and 2010. All regressions control for age (in years) and region fixed effects, age and marital status of the caregiver at birth, educational level of the caregiver (no school, primary level of education, secondary level of education and higher of education), land ownership (average percentile rank) and sex of the adolescent. Standard errors are clustered at the household level ($^{+}$ $p < 0.10$, * $p < 0.05$, ** $p < 0.01$).

Table C12: Associations between economic shocks at birth and cognitive outcomes and educational outcomes - asset ownership subset

	All (1)	Boys (2)	Girls (3)	All (4)	Boys (5)	Girls (6)
	Summary index					
	Continuous outcomes			Discrete outcomes		
1 shock at birth	0.031	0.082	-0.038	0.032	-0.033	0.099
	(0.057)	(0.079)	(0.087)	(0.067)	(0.090)	(0.105)
2 shocks or more at birth	-0.157	-0.009	-0.338*	0.303*	0.078	0.560*
	(0.110)	(0.139)	(0.169)	(0.152)	(0.186)	(0.237)
Observations	1480	748	732	1480	748	732
	A. Reading skills					
	Reading score			Can't read Chichewa		
1 shock at birth	0.139	0.250	-0.030	-0.027	-0.059	0.029
	(0.209)	(0.300)	(0.303)	(0.095)	(0.129)	(0.151)
2 shocks or more at birth	-0.381	0.034	-0.900	0.259	0.127	0.445+
	(0.377)	(0.517)	(0.557)	(0.169)	(0.237)	(0.246)
Observations	1468	742	726	1470	743	727
	B. Working memory					
	Working memory score			Score of 0		
1 shock at birth	0.041	0.162	-0.090	-0.023	-0.421+	0.413+
	(0.116)	(0.153)	(0.183)	(0.152)	(0.238)	(0.231)
2 shocks or more at birth	-0.312	-0.142	-0.498	0.516*	0.371	0.778*
	(0.211)	(0.254)	(0.321)	(0.224)	(0.312)	(0.350)
Observations	1219	610	609	1219	610	524
	C. Math skills					
	Math score			Score of 0		
1 shock at birth	-0.046	-0.025	-0.113	0.170	0.073	0.319
	(0.219)	(0.307)	(0.326)	(0.140)	(0.176)	(0.233)
2 shocks or more at birth	-0.638	-0.451	-0.932	0.264	-0.094	0.748*
	(0.404)	(0.545)	(0.588)	(0.221)	(0.311)	(0.328)
Observations	1439	727	712	1439	727	655
	D. Schooling					
	Educational attainment			Age for grade ⩾ 3		
1 shock at birth	0.015	0.119	-0.137	-0.002	-0.007	0.020
	(0.089)	(0.115)	(0.138)	(0.099)	(0.133)	(0.149)
2 shocks or more at birth	-0.115	0.215	-0.474+	0.061	-0.043	0.218
	(0.174)	(0.215)	(0.271)	(0.183)	(0.260)	(0.269)
Observations	1480	748	732	1379	698	681

Note: The sample is derived from the ACE sample collected in 2017 and 2018. Economic shocks are reported by adolescent's household as part of the MLSFH collected in 2008 and 2010. All regressions control for age (in years) and region fixed effects, age and marital status of the caregiver at birth, educational level of the caregiver (no school, primary level of education, secondary level of education and higher of education), a continuous wealth score based on a subset of assets own by the household and sex of the adolescent. Standard errors are clustered at the household level ($^{+}$ $p < 0.10$, * $p < 0.05$, ** $p < 0.01$).

Table C13: Associations between economic shocks at birth and cognitive outcomes and educational outcomes - wealth score 2004

	All (1)	Boys (2)	Girls (3)	All (4)	Boys (5)	Girls (6)
	Summary index					
	Continuous outcomes			Discrete outcomes		
1 shock at birth	0.015	0.016	-0.010	0.001	-0.037	0.004
	(0.072)	(0.096)	(0.110)	(0.084)	(0.107)	(0.142)
2 shocks or more at birth	-0.184	-0.056	-0.355	0.415^{+}	0.136	0.736^{*}
	(0.153)	(0.184)	(0.247)	(0.213)	(0.229)	(0.357)
Observations	924	454	470	924	454	470
	A. Reading skills					
	Reading score			Can't read Chichewa		
1 shock at birth	0.119	0.214	-0.105	-0.002	-0.017	0.037
	(0.264)	(0.379)	(0.381)	(0.118)	(0.160)	(0.197)
2 shocks or more at birth	-0.487	-0.335	-0.733	0.423^{+}	0.358	0.562
	(0.500)	(0.629)	(0.818)	(0.218)	(0.293)	(0.361)
Observations	918	451	467	919	452	467
	B. Working memory					
	Working memory score			Score of 0		
1 shock at birth	0.042	0.107	0.007	-0.135	-0.526^{+}	0.236
	(0.147)	(0.181)	(0.235)	(0.182)	(0.277)	(0.303)
2 shocks or more at birth	-0.339	-0.081	-0.636	0.545^{*}	0.276	0.820^{+}
	(0.295)	(0.341)	(0.439)	(0.271)	(0.375)	(0.429)
Observations	763	374	389	763	374	338
	C. Math skills					
	Math score			Score of 0		
1 shock at birth	-0.224	-0.177	-0.356	0.056	-0.183	0.238
	(0.279)	(0.391)	(0.416)	(0.173)	(0.217)	(0.289)
2 shocks or more at birth	-0.865	-0.452	-1.464^{+}	0.343	-0.072	0.906^{*}
	(0.546)	(0.703)	(0.853)	(0.276)	(0.351)	(0.424)
Observations	893	439	454	893	439	422
	D. Schooling					
	Educational attainment			Age for grade $\geqslant 3$		
1 shock at birth	-0.023	-0.015	-0.098	-0.047	0.100	-0.177
	(0.113)	(0.145)	(0.172)	(0.121)	(0.168)	(0.178)
2 shocks or more at birth	-0.192	0.010	-0.476	0.072	0.091	0.093
	(0.237)	(0.275)	(0.420)	(0.251)	(0.337)	(0.411)
Observations	924	454	470	863	426	437

Note: The sample is derived from the ACE sample collected in 2017 and 2018. Economic shocks are reported by adolescent's household as part of the MLSFH collected in 2008 and 2010. All regressions control for age (in years) and region fixed effects, age and marital status of the caregiver at birth, educational level of the caregiver (no school, primary level of education, secondary level of education and higher of education), a continuous wealth index of the household measured in 2004 and sex of the adolescent. Standard errors are clustered at the household level ($^{+}$ $p < 0.10$, * $p < 0.05$, ** $p < 0.01$).

Table C14: Associations between economic shocks at birth and cognitive outcomes - not controlling for wealth score

	All (1)	Boys (2)	Girls (3)	All (4)	Boys (5)	Girls (6)
			Summary index			
	Continuous outcomes			Discrete outcomes		
1 shock at birth	0.048	0.094	-0.021	0.016	-0.041	0.083
	(0.057)	(0.077)	(0.088)	(0.066)	(0.088)	(0.103)
2 shocks or more at birth	-0.189^{+}	-0.032	-0.372*	0.320*	0.100	0.577**
	(0.105)	(0.134)	(0.158)	(0.142)	(0.180)	(0.216)
Observations	1556	792	764	1556	792	764
			A. Reading skills			
	Reading score			Can't read Chichewa		
1 shock at birth	0.188	0.325	-0.024	-0.037	-0.075	0.042
	(0.208)	(0.291)	(0.304)	(0.092)	(0.123)	(0.146)
2 shocks or more at birth	-0.525	-0.066	-1.038*	0.313*	0.190	0.484*
	(0.362)	(0.497)	(0.522)	(0.159)	(0.231)	(0.225)
Observations	1543	786	757	1545	787	758
			B. Working memory			
	Working memory score			Score of 0		
1 shock at birth	0.060	0.158	-0.066	-0.001	-0.293	0.389^{+}
	(0.116)	(0.147)	(0.186)	(0.144)	(0.210)	(0.220)
2 shocks or more at birth	-0.337	-0.137	-0.558^{+}	0.501*	0.380	0.725*
	(0.209)	(0.250)	(0.316)	(0.218)	(0.306)	(0.341)
Observations	1277	644	633	1277	644	545
			C. Math skills			
	Math score			Score of 0		
1 shock at birth	0.027	0.082	-0.088	0.131	0.018	0.324
	(0.218)	(0.302)	(0.324)	(0.136)	(0.173)	(0.228)
2 shocks or more at birth	-0.652^{+}	-0.501	-0.910^{+}	0.224	-0.148	0.759*
	(0.386)	(0.531)	(0.552)	(0.213)	(0.309)	(0.314)
Observations	1512	770	742	1512	770	680
			D. Schooling			
	Educational attainment			Age for grade $\geqslant$ 3		
1 shock at birth	0.056	0.165	-0.100	-0.041	-0.055	-0.008
	(0.089)	(0.115)	(0.136)	(0.096)	(0.129)	(0.145)
2 shocks or more at birth	-0.156	0.164	-0.470^{+}	0.146	0.025	0.327
	(0.166)	(0.212)	(0.256)	(0.178)	(0.259)	(0.256)
Observations	1556	792	764	1449	738	711

Note: The sample is derived from the ACE sample collected in 2017 and 2018. Economic shocks are reported by adolescent's household as part of the MLSFH collected in 2008 and 2010. All regressions control for age (in years) and region fixed effects, age and marital status of the caregiver at birth, educational level of the caregiver (no school, primary level of education, secondary level of education and higher of education) and sex of the adolescent. Note that wealth score is not controlled for in these estimations. Standard errors are clustered at the household level ($^{+}$ $p < 0.10$, * $p < 0.05$, ** $p < 0.01$).

D Possible mechanisms

Anthropometrics

Early-life physical development is an important determinant of later-life cognitive outcomes. The gender differences we find could therefore stem from the fact that economic shocks at birth impact the physical development of girls differently from that of boys in the early years of life, leading to differences in cognitive and educational outcomes in adolescence. However, Table D1 shows that adolescents who experience economic shocks during the year of their birth do not appear to have different anthropometric characteristics as measured by height and height z-scores[6], where height is generally used as a proxy for stress exposure and deprivation experienced in utero and early in life (Beach *et al.* 2018; Currie and Vogl 2013; Parman 2015; Thomas *et al.* 1990; WHO 1995). Note that this holds true for both girls and boys. For a sub-sample of adolescents, we also have measures for their height in early childhood, as part of data collected for the 2008 MLSFH survey. We do not find any statistically significant associations between economic shocks during the year of birth and adolescent's height and height z-scores[7] measured during early childhood in 2008 (Table D2). Our results indicate that adolescent's height and height z-score are not affected by economic shocks experienced at birth. We therefore find no evidence for a biological impact using height. This is however a rough marker for biological development and may not be adequate to capture more subtle physiological changes.

Investment in schooling

We start our analysis by looking closely at the importance of early investment in education. Adolescents who live in households that experience economic shocks during the year of birth might receive lower investment in education, which could result in lower cognitive and educational outcomes later in life. The 2010 MLSFH survey contains a module on investment in education in which respondents were asked how much they have invested in the investment of children in their household who were between 5 and 25 years of age. More specifically, respondents were asked how much they spent in school fees and school supplies for the children in their household over the past year. We merge this information to adolescents in our sample to assess to what

[6]The z-score is derived using the characteristics of the height distribution in our sample of adolescents. It is sex and age (in years) specific. We cannot use the WHO growth standards to compute the z-score because these standards exist only for children up to 5 years of age.

[7]The z-score is derived using the WHO standard height characteristics, which are available only for children up to 5 years old.

extent their experience of a negative economic shock at birth affected the investment in education of their caregiver in 2010. Note that the following analysis is restricted to adolescents who were born in 2005 and before, which results in a substantial reduction in the size of our sample.

As detailed below, we find suggestive evidence that investment in education could be the reason why we observe negative associations between economic shocks at birth and cognitive outcomes and educational attainment for girls but not for boys. We find evidence that these gender differences possibly stem from changes in investment in education, where boys appear to be relatively protected from cuts in investment whereas girls suffer from investment cuts following negative economic shocks that occur during the year of their birth.

Table D3 presents the associations between experiencing an economic shock at birth and educational investment.[8] Because of the nature of our dependent variable, we estimate two-part models to account for the large number of 0's and the substantial skewness in the distribution, which are typical in expenditure data. We specify our two-part model by choosing a probit specification for the extensive margin analysis, a log transform for the link function[9] and a gamma distribution to define the variance as being proportional to the square of the mean of our outcome variable.[10] The results of the associations between economic shocks and investment in education, both at the extensive (probit) and intensive margin (GLM) are presented in Table D3. The outcome variable in the first three columns corresponds to the total amount that was invested in education in 2010 for a particular adolescent in the ACE sample. Columns 4 to 6 and 7 to 9 break this total down by whether the amount is coming from the household or by someone else outside the household. [11] Note that in this

[8]Again, because of the small sample size, we are unable to differentiate between adolescents who experienced one or more shocks at birth and hence present results in which these two categories are combined.

[9]In our benchmark specification, the θ coefficient of a Box-Cox approach test is equal to 0.131, which is close to 0 and hence corresponds to the natural log transform (Deb and Norton 2018). The result is very similar when we do not control for any covariates in the specification.

[10]We follow Deb and Norton (2018) and proceed to a modified Park test (Park 1966) that consists in estimating a GLM (with log link and gamma distribution) from which we derive the conditional expected mean and squared error term for each observation. We then regress the squared error term on conditional expected mean and look at the value of that coefficient to determine the most appropriate distribution to use. In our benchmark specification, the coefficient associated with that regression equals 2.220, and we cannot reject the hypothesis that the coefficient is equal to 2 (p-value=0.680), which suggests the use of a gamma distribution (Deb and Norton 2018).

[11]Unfortunately, we do not know the relationship between these other donors/sponsors and the adolescents.Moreover, the amount of money coming from outside the household is small relative to investment coming from the household. Out of the 692 adolescents in the sample under consideration,

specification we control for the total number of children present in the household, the total number of girls, the age order and the number of children that are reported in the educational investment module of the 2010 MLSFH survey. Column 3 of Table D3 shows that girls who experienced a negative economic shock during the year of birth received lower educational investment from their households compared to girls who did not experience any shocks at birth. The lower investment is coming from the intensive margin, meaning that girls who experienced a shock at birth were not more or less likely to receive a positive amount, but the amount they were receiving was significantly lower than others. We do not observe corresponding associations for boys (Column 2). When examining where the money is coming from, we observe that it is mostly the investment in education coming from persons outside the household that reduce the investment on girls—both at the intensive and extensive margins—whereas the household itself does not seem to change its investment in education behavior after an economic shock.

Tables D4 and D5 show that it is economic shocks at birth that affect investment in education later in life, and that economic shocks that occur two years after the year of birth do not lead to similar associations. This is surprising given that shocks that occur two years after the year of birth are temporally closer to when decisions about investment in schooling are made.

Moreover, our analysis suggests the presence of some compensatory behaviors in terms of investment in education for boys. Table D6 shows that economic shocks that occur during the year of birth of a boy not only increase mean investment in education for boys within the household, but that economic shocks during the year of birth of girls increase mean investment on boys as well.

Furthermore, we show that investment in education at the household level predicts the cognitive and educational outcomes of the adolescents in our sample. Table D7 analyzes the associations between investment on schooling (at the household level) and cognitive outcomes, when considering investment in education as a continuous measure using a inverse hyperbolic sine transformation.[12] This table suggests that higher investment in education at the household level appears to be particularly beneficial for girls and less so for boys. This is consistent with the results from the previous

only 14% of them receive money for their education from someone outside their household. Note also that 38% of them do not receive any money at all (from both their own household and someone outside the household). The ratio of the amount received from someone outside the household over the total amount invested on the children's education is equal to 0.11. This means that among adolescents who receive a positive amount of investment for their education, about 11% of it is coming from someone outside their household on average.

[12] This transformation is roughly similar to the natural logarithm but allows for 0 (specifically, the transformation approaches $\ln(x+1)$ for small values of X, and $\ln(x)$ for large values of x).

table: girls outcomes are more sensitive to investment in education, and economic shocks decrease the amount of investment that is spent on their own education.[13]

Finally, we test whether economic shocks at birth have changed caregivers' perception about gender. Our analysis suggests gendered response to economic shocks for investment in education and we therefore explore whether we obtain similar effects for gender attitudes more broadly.

To do so, we exploit the 2008 MLSFH "Attitudinal Questions" module. In this module, MLSFH respondents (caregivers of the adolescents) are asked various questions about gender attitudes. More specifically, the questions ask respondents whether it is proper for a wife to leave her husband under various circumstances, such as if he beats her frequently and does not support her and the children financially. There are in total nine different scenarios that are presented to the respondents. The module also asks four questions that are related to sexual attitudes, such as whether a woman has the right to refuse unprotected sex with her husband when she thinks her husband may have HIV/AIDS or whether it is acceptable for the husband to sleep with another sexual partner if a women often refuses sex with her husband. Possible answers to these questions are Yes (1) or No (0) From these questions, we derived four different measures of "attitudes towards gender". The first one takes the sum of the "Yes" to the first nine questions (excluding sexual attitudes). The second measures takes the sum of the "Yes" to all items, including sexual attitudes. The reason we differentiate the two is because sexual attitudes might be different from more general gender attitudes. The first outcome therefore corresponds to a "gender attitude" score that ranges from 0 to 9 and the second one ranges from 0 to 13. To mirror the analysis conducted in the main text, we follow Anderson (2008) and also created two summary indices using these two sets of items (one with questions 1-9 and another with questions 1-13). If economic shocks were to change attitude towards gender, we would expect a negative association between economic shocks at birth and these various measures of gender attitudes. Table D8 shows however that economics shocks at birth do not seem to be associated with attitudes towards gender measured in 2008. The first panel shows the results when restricting the outcome variables to non-sexual attitudes, and the second panels includes all the items. The first three columns show the results when we consider the total score and the last three columns consider the summary index instead. None of the associations is statistically significant at conventional statistical level ($p < 0.05$). Moreover, when looking at the item separately, out of the 39

[13]Ideally, one would want to assess the effects of economic shocks at birth on investment in education for a particular child and consequently their effects on cognitive and educational attainment. It is however not possible to do so in our sample given that we have information about investment in education only for adolescents who are born prior to 2005.

regressions we run (13 items times 3 (all, boys and girls)), none of the coefficients associated to "two or more shocks at birth" are statistically significant at conventional level ($p < 0.05$) and only two are statistically significant at 90% confidence and no clear pattern emerges from these regressions. Economic shocks at birth therefore do not appear to be associated with short term change in gender attitudes. Our results therefore points to possible change in gender attitudes that are restricted to education and schooling investment, but not more broadly.

Another way to test this hypothesis would have been to restrict our analysis to families with only boys or girls and assess whether we obtain similar associations to those presented in the main text. If that were the case, then we could have ruled out a change in gender attitudes that are restricted to education and schooling investment altogether. That being said, fertility rate is very high in rural Malawi and most households have both boys and girls, which prevents us from testing this hypothesis using the data at hand.

Table D1: Associations between economic shocks at birth and anthropometric characteristics of the adolescents measured in 2017/2018

	Height in cm			Height z-score		
	All (1)	Boys (2)	Girls (3)	All (4)	Boys (5)	Girls (6)
1 shock at birth	0.463	0.066	0.953	0.080	0.020	0.141
	(0.504)	(0.661)	(0.764)	(0.072)	(0.091)	(0.110)
2 shocks or more at birth	0.647	0.447	1.014	0.094	0.080	0.143
	(0.837)	(1.159)	(1.193)	(0.122)	(0.167)	(0.177)
Observations	1499	767	732	1499	767	732

Note: The sample is derived from the ACE sample collected in 2017 and 2018. Economic shocks are reported by adolescent's household as part of the MLSFH collected in 2008 and 2010. All regressions control for age (in years) and region fixed effects, age and marital status of the caregiver at birth, educational level of the caregiver (no school, primary level of education, secondary level of education and higher of education), a continuous wealth index of the household and sex of the adolescent. Standard errors are clustered at the household level ($^{+}$ $p < 0.10$, * $p < 0.05$, ** $p < 0.01$).

Table D2: Associations between economic shocks at birth and anthropometric characteristics of the adolescents measured in 2008

	Height in cm			Height z-score		
	All (1)	Boys (2)	Girls (3)	All (4)	Boys (5)	Girls (6)
Shock at birth	-0.940	-0.706	-0.766	-0.175	-0.115	-0.141
	(0.750)	(0.990)	(1.232)	(0.226)	(0.309)	(0.355)
Observations	313	157	156	313	157	156

Note: The sample is derived from the ACE sample collected in 2017 and 2018 and match to information collected in 2008 as part of the 2008 MLSFH survey. Economic shocks are reported by adolescent's household as part of the MLSFH collected in 2008 and 2010. All regressions control for age (in years), month of birth and region fixed effects, age and marital status of the caregiver at birth, educational level of the caregiver (no school, primary level of education, secondary level of education and higher of education), a continuous wealth index of the household and sex of the adolescent. Standard errors are clustered at the household level ($^{+}$ $p < 0.10$, * $p < 0.05$, ** $p < 0.01$).

Table D3: **Associations between economic shocks and investment in education**

	Total			Household			Others		
	All	Boys	Girls	All	Boys	Girls	All	Boys	Girls
	(1)	(2)	(3)	(4)	(5)	(6)	(7)	(8)	(9)
Extensive margin (Probit)									
Shock at birth	0.020	-0.061	0.138	-0.009	-0.101	0.114	-0.179	0.090	-0.615^{+}
	(0.182)	(0.235)	(0.265)	(0.181)	(0.233)	(0.265)	(0.206)	(0.271)	(0.340)
Intensive margin (GLM)									
Shock at birth	-0.144	0.328	-0.417^{+}	-0.037	0.212	-0.196	-0.618	0.500	-1.436*
	(0.170)	(0.249)	(0.224)	(0.172)	(0.263)	(0.218)	(0.467)	(0.744)	(0.578)
Observations	692	333	359	694	334	360	694	333	361

Note: The sample is derived from the ACE sample collected in 2017 and 2018 for which we have information about the amount that were spent for their schooling. Economic shocks are reported by adolescent's household as part of the MLSFH collected in 2008 and 2010. All regressions control for age (in years) and region fixed effects, age and marital status of the caregiver at birth, educational level of the caregiver (no school, primary level of education, secondary level of education and higher of education), a continuous wealth index of the household, sex of the adolescent, total number of children present in the household, the total number of girls, the age order and the number of children that are reported in the educational investment module of the 2010 MLSFH survey. Standard errors are clustered at the household level ($^{+}$ $p < 0.10$, * $p < 0.05$, ** $p < 0.01$).

Table D4: Associations between economic shocks and investment in education - including only shocks that occurred 2 years after the year of birth

	Total			Household			Others		
	All	Boys	Girls	All	Boys	Girls	All	Boys	Girls
	(1)	(2)	(3)	(4)	(5)	(6)	(7)	(8)	(9)
Extensive margin (Probit)									
Shock 2 years after birth	0.012	0.113	-0.122	0.049	0.163	-0.084	-0.027	-0.067	0.037
	(0.124)	(0.168)	(0.180)	(0.123)	(0.163)	(0.179)	(0.141)	(0.204)	(0.194)
Intensive margin (GLM)									
Shock 2 years after birth	-0.030	0.130	-0.147	-0.077	0.110	-0.234	-0.005	0.341	0.259
	(0.112)	(0.151)	(0.155)	(0.111)	(0.151)	(0.158)	(0.309)	(0.407)	(0.368)
Observations	692	333	359	694	334	360	694	333	361

Note: The sample is derived from the ACE sample collected in 2017 and 2018 for which we have information about the amount that were spent for their schooling. Economic shocks are reported by adolescent's household as part of the MLSFH collected in 2008 and 2010. All regressions control for age (in years) and region fixed effects, age and marital status of the caregiver at birth, educational level of the caregiver (no school, primary level of education, secondary level of education and higher of education), a continuous wealth index of the household, sex of the adolescent, total number of children present in the household, the total number of girls, the age order and the number of children that are reported in the educational investment module of the 2010 MLSFH survey. Standard errors are clustered at the household level ($^{+}$ $p < 0.10$, * $p < 0.05$, ** $p < 0.01$).

Table D5: Associations between economic shocks and investment in education - including also shocks that occurred 2 years after the year of birth

	Total			Household			Others		
	All (1)	Boys (2)	Girls (3)	All (4)	Boys (5)	Girls (6)	All (7)	Boys (8)	Girls (9)
Extensive margin (Probit)									
Shock at birth	0.023	-0.047	0.101	0.001	-0.081	0.090	-0.187	0.082	-0.616^{+}
	(0.183)	(0.238)	(0.262)	(0.182)	(0.235)	(0.263)	(0.209)	(0.270)	(0.344)
Shock 2 years after birth	0.014	0.110	-0.106	0.049	0.157	-0.070	-0.042	-0.061	-0.006
	(0.124)	(0.169)	(0.179)	(0.124)	(0.163)	(0.177)	(0.144)	(0.203)	(0.200)
Intensive margin (GLM)									
Shock at birth	-0.154	0.347	-0.475*	-0.051	0.223	-0.259	-0.625	0.740	-1.355*
	(0.174)	(0.253)	(0.225)	(0.173)	(0.265)	(0.215)	(0.470)	(0.925)	(0.677)
Shock 2 years after birth	-0.044	0.149	-0.200	-0.081	0.118	-0.257	-0.042	0.465	0.143
	(0.113)	(0.152)	(0.158)	(0.112)	(0.152)	(0.158)	(0.304)	(0.501)	(0.396)
Observations	692	333	359	694	334	360	694	333	361

Note: The sample is derived from the ACE sample collected in 2017 and 2018 for which we have information about the amount that were spent for their schooling. Economic shocks are reported by adolescent's household as part of the MLSFH collected in 2008 and 2010. All regressions control for age (in years) and region fixed effects, age and marital status of the caregiver at birth, educational level of the caregiver (no school, primary level of education, secondary level of education and higher of education), a continuous wealth index of the household, sex of the adolescent, total number of children present in the household, the total number of girls, the age order and the number of children that are reported in the educational investment module of the 2010 MLSFH survey. Standard errors are clustered at the household level ($^{+}$ $p < 0.10$, * $p < 0.05$, ** $p < 0.01$).

Table D6: Associations between economic shocks at birth and investment in education at the household level

	**Mean schooling investment on boys in HH**		**Mean schooling investment on girls in HH**	
	Shock on boys (1)	Shock on girls (2)	Shock on boys (3)	Shock on girls (4)
Extensive margin (Probit)				
Shock at birth	0.028	0.466*	0.260	0.105
	(0.202)	(0.226)	(0.187)	(0.161)
Intensive margin (GLM)				
Shock at birth	0.264^{+}	-0.014	0.206	0.141
	(0.148)	(0.157)	(0.163)	(0.159)
Observations	431	403	480	562

Note: The sample is derived from the ACE sample collected in 2017 and 2018 for which we have information about the amount spent on education at the household level. Economic shocks are reported by adolescent's household as part of the MLSFH collected in 2008 and 2010. All regressions control for age (in years) and region fixed effects, age and marital status of the caregiver at birth, educational level of the caregiver (no school, primary level of education, secondary level of education and higher of education), a continuous wealth index of the household, sex of the adolescent, total number of children present in the household, the total number of girls, the number of children that are reported in the educational investment module of the 2010 MLSFH survey as well as their average age and the one of the girls in the module. Standard errors are clustered at the household level ($^{+}$ $p < 0.10$, * $p < 0.05$, ** $p < 0.01$).

Table D7: Associations between investment in education and cognitive and educational attainment outcomes

	All (1)	Boys (2)	Girls (3)	All (4)	Boys (5)	Girls (6)
	Summary index					
	Continuous outcomes			Discrete outcomes		
Investment in education (ihst)	0.028**	0.025+	0.031*	-0.022*	-0.012	-0.033*
	(0.010)	(0.014)	(0.013)	(0.011)	(0.015)	(0.013)
Observations	1632	819	813	1632	819	813
	A. Reading skills					
	Reading score			Can't read Chichewa		
Investment in education (ihst)	0.093**	0.069	0.113*	-0.034*	-0.017	-0.054**
	(0.036)	(0.049)	(0.045)	(0.016)	(0.021)	(0.021)
Observations	1616	812	804	1612	809	803
	B. Working memory					
	Working memory score			Score of 0		
Investment in education (ihst)	-0.005	0.001	-0.011	-0.004	0.017	-0.028
	(0.022)	(0.028)	(0.028)	(0.024)	(0.032)	(0.032)
Observations	1317	654	663	1310	650	660
	C. Mathematical skills					
	Math score			Score of 0		
Investment in education (ihst)	0.095*	0.070	0.121*	-0.023	-0.022	-0.033
	(0.038)	(0.053)	(0.047)	(0.020)	(0.026)	(0.027)
Observations	1590	797	793	1583	793	715
	D. Schooling					
	Educational attainment			Age for grade $\geqslant 3$		
Investment in education (ihst)	0.080**	0.071**	0.090**	-0.043*	-0.027	-0.062**
	(0.016)	(0.022)	(0.020)	(0.018)	(0.025)	(0.023)
Observations	1632	819	813	1494	760	734

Note: The sample is derived from the ACE sample collected in 2017 and 2018 for which we have information about the amount spent on education by their household. All regressions control for age (in years) and region fixed effects, age and marital status of the caregiver at birth, educational level of the caregiver (no school, primary level of education, secondary level of education and higher of education), a continuous wealth index of the household and sex of the adolescent. We also control for the number of children living in the household. "ihst" stands for inverse hyperbolic sine transformation. Standard errors are clustered at the household level ($^{+}$ $p < 0.10$, * $p < 0.05$, ** $p < 0.01$).

Table D8: Associations between attitudes towards gender and economic shocks

	All<br>(1)	Boys<br>(2)	Girls<br>(3)	All<br>(4)	Boys<br>(5)	Girls<br>(6)
	More liberal gender attitude					
	Total score			Summary index		
1 shock at birth	0.025	-0.122	0.203	0.030	-0.045	0.118
	(0.120)	(0.166)	(0.167)	(0.063)	(0.087)	(0.091)
2 shocks at birth	-0.057	-0.318	0.150	-0.022	-0.142	0.075
	(0.217)	(0.325)	(0.288)	(0.111)	(0.159)	(0.154)
Observations	1544	787	757	1544	787	757
	More liberal gender attitude including sexual practices					
	Total score			Summary index		
1 shock at birth	0.144	0.036	0.283	0.086	0.029	0.156^{+}
	(0.138)	(0.191)	(0.193)	(0.066)	(0.090)	(0.093)
2 shocks at birth	-0.080	-0.263	0.059	-0.048	-0.129	0.015
	(0.255)	(0.391)	(0.339)	(0.116)	(0.176)	(0.156)
Observations	1544	787	757	1544	787	757

Note: The sample is derived from the ACE sample collected in 2017 and 2018. Economic shocks are reported by adolescent's household as part of the MLSFH collected in 2008 and 2010. All regressions control for age (in years) and region fixed effects, age and marital status of the caregiver at birth, educational level of the caregiver (no school, primary level of education, secondary level of education and higher of education), a continuous wealth index of the household and sex of the adolescent. Standard errors are clustered at the household level ($^{+}$ $p < 0.10$, * $p < 0.05$, ** $p < 0.01$). "Total score" is derived by taking the sum of the different "gender attitudes" items and "Summary index" corresponds to the weighted index of the same items.

Table D9: Indirect tests for mortality selection

	Female OLS (1)	Female OLS (2)	Female Probit (3)	Female Probit (4)
1 shock at birth	-0.049	-0.028	-0.124	-0.070
	(0.033)	(0.035)	(0.082)	(0.087)
2 shocks or more at birth	0.012	0.074	0.031	0.190
	(0.058)	(0.061)	(0.145)	(0.155)
Age: 11 years old		-0.076		-0.194
		(0.062)		(0.159)
Age: 12 years old		0.009		0.024
		(0.062)		(0.155)
Age: 13 years old		0.063		0.158
		(0.062)		(0.155)
Age: 14 years old		0.016		0.039
		(0.061)		(0.153)
Age: 15 years old		0.061		0.153
		(0.066)		(0.167)
Age of the caregiver at birth		-0.000		-0.000
		(0.001)		(0.003)
South region		0.033		0.084
		(0.032)		(0.081)
North region		0.036		0.090
		(0.036)		(0.090)
Caregiver married at birth		0.010		0.027
		(0.038)		(0.097)
Primary level education - caregiver		0.020		0.051
		(0.033)		(0.083)
Secondary level education - caregiver		-0.015		-0.039
		(0.058)		(0.147)
Wealth score		0.010		0.026
		(0.007)		(0.019)
Observations	1559	1556	1559	1556

Note: Standard errors in parentheses clustered at the household level ($^{+}$ $p < 0.10$, * $p < 0.05$, ** $p < 0.01$). The coefficients are the results of linear regressions for which the outcome variable is a dichotomous variable that takes the values 1 if the adolescent is a girl. The sample is derived from the ACE sample collected in 2017 and 2018. Economic shocks are reported by adolescent's household as part of the MLSFH collected in 2008 and 2010.

References

Anderson, M. L. (2008). Multiple inference and gender differences in the effects of early intervention: A reevaluation of the abecedarian, perry preschool, and early training projects. *Journal of the American statistical Association*, **103**(484), 1481–1495.

Beach, B., Ferrie, J. P., and Saavedra, M. H. (2018). Fetal shock or selection? The 1918 Influenza pandemic and human capital development. Technical report, National Bureau of Economic Research.

Currie, J. and Vogl, T. (2013). Early-life health and adult circumstance in developing countries. *Annu. Rev. Econ.*, **5**(1), 1–36.

Deb, P. and Norton, E. C. (2018). Modeling health care expenditures and use. *Annual Review of Public Health*, **39**, 489–505.

Park, R. E. (1966). Estimation with heteroscedastic error terms. *Econometrica (pre-1986)*, **34**(4), 888.

Parman, J. (2015). Childhood health and human capital: New evidence from genetic brothers in arms. *The Journal of Economic History*, **75**(1), 30–64.

Thomas, D., Strauss, J., and Henriques, M.-H. (1990). Child survival, height for age and household characteristics in Brazil. *Journal of Development Economics*, **33**(2), 197–234.

UNICEF (2020). Girls' education. `https://www.unicef.org/education/girls-education`. Accessed: 2020-09-21.

WHO (1995). The use and interpretation of anthropometry. Report 8854, World Health Organization.
